# Whole-genome analysis to determine the rate and patterns of intra-subtype reassortment among influenza type-A viruses in Africa

**DOI:** 10.1093/ve/veac005

**Published:** 2022-01-29

**Authors:** Grace Nabakooza, Andrzej Pastusiak, David Patrick Kateete, Julius Julian Lutwama, John Mulindwa Kitayimbwa, Simon David William Frost

**Affiliations:** Department of Immunology and Molecular Biology, Makerere University, Old Mulago Hill Road, P.O Box 7072, Kampala, Uganda; UVRI Centre of Excellence in Infection and Immunity Research and Training (MUII-Plus), Makerere University, Plot No: 51-59 Nakiwogo Road, P.O. Box 49, Entebbe, Uganda; Centre for Computational Biology, Uganda Christian University, Plot 67-173, Bishop Tucker Rd, P.O BOX 4, Mukono, Uganda; Microsoft Research, 14820 NE 36th Street, Redmond, WA 98052, USA; Department of Immunology and Molecular Biology, Makerere University, Old Mulago Hill Road, P.O Box 7072, Kampala, Uganda; UVRI Centre of Excellence in Infection and Immunity Research and Training (MUII-Plus), Makerere University, Plot No: 51-59 Nakiwogo Road, P.O. Box 49, Entebbe, Uganda; Department of Arbovirology Emerging & Re-Emerging Infectious Diseases, Uganda Virus Research Institute (UVRI), Plot No: 51-59, Nakiwogo Road, P.O. Box 49, Entebbe, Uganda; UVRI Centre of Excellence in Infection and Immunity Research and Training (MUII-Plus), Makerere University, Plot No: 51-59 Nakiwogo Road, P.O. Box 49, Entebbe, Uganda; Centre for Computational Biology, Uganda Christian University, Plot 67-173, Bishop Tucker Rd, P.O BOX 4, Mukono, Uganda; Microsoft Research, 14820 NE 36th Street, Redmond, WA 98052, USA; London School of Hygiene & Tropical Medicine (LSHTM), Keppel St, Bloomsbury, London WC1E 7HT, UK

**Keywords:** influenza A, H1N1pdm09, H3N2, whole-genome sequencing, genomics, evolution, reassortment, intra-subtype reassortment, phylogenetics, transmission, Uganda, Africa

## Abstract

Influenza type-A viruses (IAVs) present a global burden of human respiratory infections and mortality. Genome reassortment is an important mechanism through which epidemiologically novel influenza viruses emerge and a core step in the safe reassortment-incompetent live-attenuated influenza vaccine development. Currently, there are no data on the rate, spatial and temporal distribution, and role of reassortment in the evolution and diversification of IAVs circulating in Africa. We aimed to detect intra-subtype reassortment among Africa pandemic H1N1pdm09 (2009–10), seasonal H1N1pdm09 (2011–20), and seasonal H3N2 viruses and characterize the genomic architecture and temporal and spatial distribution patterns of the resulting reassortants. Our study was nested within the Uganda National Influenza Surveillance Programme. Next-generation sequencing was used to generate whole genomes (WGs) from 234 H1N1pdm09 (*n* = 116) and H3N2 (*n* = 118) viruses sampled between 2010 and 2018 from seven districts in Uganda. We combined our newly generated WGs with 658 H1N1pdm09 and 1131 H3N2 WGs sampled between 1994 and 2020 across Africa and identified reassortants using an automated Graph Incompatibility Based Reassortment Finder software. Viral reassortment rates were estimated using a coalescent reassortant constant population model. Phylogenetic analysis was used to assess the effect of reassortment on viral genetic evolution. We observed a high frequency of intra-subtype reassortment events, 12 · 4 per cent (94/758) and 20 · 9 per cent (256/1,224), and reassortants, 13 · 3 per cent (101/758) and 38 · 6 per cent (472/1,224), among Africa H1N1pdm09 and H3N2 viruses, respectively. H1N1pdm09 reassorted at higher rates (0.1237–0.4255) than H3N2 viruses (0 · 00912–0.0355 events/lineage/year), a case unique to Uganda. Viral reassortants were sampled in 2009 through 2020, except in 2012. 78 · 2 per cent (79/101) of H1N1pdm09 reassortants acquired new non-structural, while 57 · 8 per cent (273/472) of the H3N2 reassortants had new hemagglutinin (H3) genes. Africa H3N2 viruses underwent more reassortment events involving larger reassortant sets than H1N1pdm09 viruses. Viruses with a specific reassortment architecture circulated for up to five consecutive years in specific countries and regions. The Eastern (Uganda and Kenya) and Western Africa harboured 84 · 2 per cent (85/101) and 55 · 9 per cent (264/472) of the continent’s H1N1pdm09 and H3N2 reassortants, respectively. The frequent reassortment involving multi-genes observed among Africa IAVs showed the intracontinental viral evolution and diversification possibly sustained by viral importation from outside Africa and/or local viral genomic mixing and transmission. Novel reassortant viruses emerged every year, and some persisted in different countries and regions, thereby presenting a risk of influenza outbreaks in Africa. Our findings highlight Africa as part of the global influenza ecology and the advantage of implementing routine whole-over partial genome sequencing and analyses to monitor circulating and detect emerging viruses. Furthermore, this study provides evidence and heightens our knowledge on IAV evolution, which is integral in directing vaccine strain selection and the update of master donor viruses used in recombinant vaccine development.

## 1. Introduction

Influenza type-A viruses (IAVs) repeatedly undergo genetic changes resulting in novel viruses that cause seasonal epidemics and pandemics worldwide (Bouvier and Palese 2008). Genetic characterization of IAVs to understand how novel viruses emerge and spread between locations over time is vital for effective viral control and prevention.

IAVs carry enveloped genomes consisting of eight gene segments of single-stranded ribonucleic acid (RNA) molecules (Bouvier and Palese 2008). The segmented nature of the genomes allows for gene exchanges between virions co-infecting a cell during replication, a process termed reassortment, reviewed here (McDonald et al. 2016).

Four past global influenza pandemics: the 1918-H1N1, 1957-H2N2, 1968-H3N2, and the 2009-H1N1 (H1N1pdm09) swine flu resulted from gene exchanges between avian and swine influenza viruses (inter-subtype reassortment), killing millions of people (Taubenberger and Morens 2010; McDonald et al. 2016). For example, the H1N1pdm09 pandemic killed approximately 500,000 people globally (Dawood et al. 2012). The effects of the pandemics are further exacerbated by seasonal influenza infections. The latter account for excess mortality at a rate of 4 · 0–8 · 8 per 100,000 individuals globally, with Sub-Saharan Africa having the highest rates ranging from 2 · 8 to 16 · 5 per 100,000 individuals (Iuliano, Roguski, and Chang 2018).

Gene exchanges among the same hemagglutinin–neuraminidase (HA-NA) subtype viruses (intra-subtype reassortment) were reported among H1N1 and H3N2 viruses, resulting in short-term (Nelson et al. 2006), antigenically novel (Rambaut et al. 2008), highly transmissible (Nelson et al. 2008), and drug-resistant (Simonsen et al. 2007) reassortant viruses.

Phylogenetic-based methods (PBMs) have been widely applied on whole genomes (WGs) to detect both inter-and intra-subtype reassortment of IAVs sampled in Europe, Asia, and the USA (Lindstrom, Cox, and Klimov 2004; Ghedin et al. 2005; Holmes et al. 2005; Nelson et al. 2008, 2012; Rambaut et al. 2008; Westgeest, Russell, and Lin 2014). PBMs assume the absence of reassortment if any two gene sequences from the same virus occupy similar positions in their respective phylogenies (total congruence). When the two sequences occupy conflicting phylogenetic positions (incongruence), it indicates their different origins (Nelson and Holmes 2007). Studies have shown that reassortment frequency and duration of reassortant circulation varies between viral subtypes (Lindstrom, Cox, and Klimov 2004; Ghedin et al. 2005; Holmes et al. 2005; Nelson et al. 2008, 2012; Rambaut et al. 2008; Westgeest, Russell, and Lin 2014). A study by Berry et al. reported a steady frequency (3 · 35 per cent) of reassortment events among H3N2 viruses independent of previous viral evolution and sample location (Maljkovic Berry et al. 2016). Another study by Müller et al. used a network-based coalescent reassortant constant population model on viral sequences sampled from Europe, America, and Asia. The model estimated high reassortment rates among the 1995–2019 H3N2 and 2009 H1N1pdm09 viruses at 0 · 35–0 · 65 and 0 · 15–0 · 8 events/lineage/year, respectively (Müller et al. 2020).

Influenza epidemics in Africa usually involve the co-circulation of multiple viral lineages and subtypes all-year-round, with distinct influenza peaks in North and South Africa (Radin et al. 2012), which might enhance viral reassortment. Unfortunately, there is no exhaustive information on intra-subtype reassortment and its role in the genetic evolution of IAVs in Africa. With this dearth of knowledge about drivers of Africa IAVs evolution, the continent remains to benefit minimally from potential influenza control strategies.

We aimed to identify reassortment events and reassortant viruses involved in these events among Africa H1N1pdm09 and H3N2 viruses and characterize the temporal and spatial distribution of reassortants from 1994 to 2020 in Africa. Our approach enabled us to estimate the frequency of reassortment among Africa IAVs, which we compared to the coalescent-based reassortment rate (Müller et al. 2020) and identified African hubs for influenza reassortment.

## 2. Materials and methods

### 2.1 Study area, population, and sampling

**Figure 1.**
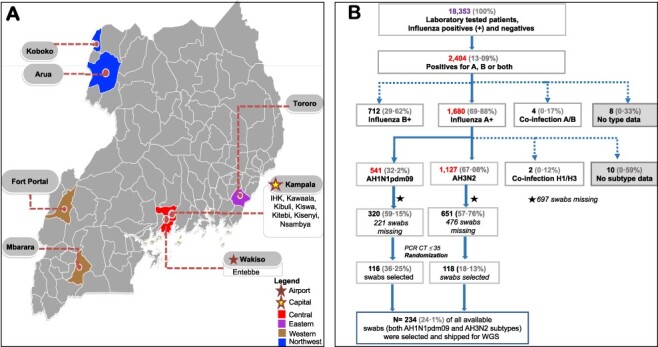
Geographical distribution of sampled sentinel sites and selection of patients’ swabs for influenza whole-genome sequencing. Panel A shows the distribution of sampled sentinel sites in the UVRI-NIC influenza surveillance programme. District hospitals included Arua-ARU, Entebbe-EBB, Fort Portal-FTL, Koboko-KBK, Mbarara-MBA, Tororo-TOR, and Kampala. Kampala had three hospitals: International Hospital Kampala-IHK, Kibuli moslem hospital-KIB, Nsambya Hospital-NSY, and four clinics: Kawaala Health Center-KIS, Kiswa Health Center-KSW, Kisenyi Health Center-KSY, and Kitebi health centre-KIS samples. Swabs were assigned unique laboratory identification numbers using the site codes and a number. For example, EBB0001 for swab 0001 from Entebbe. Panel B is a flowchart showing the exclusion and inclusion criteria for patients’ swab samples selected for influenza whole-genome sequencing (WGS). Patients diagnosed with either influenza subtypes H1N1pdm09 or H3N2 and whose swabs had a PCR CT ≤ 35 had their laboratory codes randomised based on the subtype and year of collection using the R software v3.6.3 (https://www.r-project.org For years with few swabs (*n* < 15), all available swabs were retrieved. The missing swabs (*n* = 697) consisted of some shipped to the CDC for routine surveillance and others lost due to accidental failure of a freezer. The numbers are based on the UVRI-NIC laboratory dataset only, as of 9 May 2018.

The National Influenza Centre at the Uganda Virus Research Institute (UVRI-NIC) implements clinic-and hospital-based influenza surveillance in 13 peri-urban and densely populated sites in seven districts across Uganda (Lutwama et al. 2012) (Fig. 1A).

Nasal and oropharyngeal swabs collected from patients of all ages presenting with influenza-like illnesses (ILIs) and severe acute respiratory infections (SARI) were screened for influenza A and B. The influenza A positives were subtyped as H1N1, H1N1pdm09, and H3N2 using the Centers for Disease Control and Prevention’s (CDC) real-time reverse-transcription polymerase chain reaction (rRT-PCR) protocols and primers (Atlanta, Georgia) (C.D.C. 2009). All available H1N1pdm09 and H3N2 patients’ swabs with PCR cycle threshold (CT ≤ 35) were stratified by subtype and year of collection and randomly selected every first 15 swabs from each stratum. A total of 234; 116 H1N1pdm09 and 118 H3N2 patients’ swabs were selected for WG sequencing (Fig. 1B).

### 2.2 Virus RNA isolation, next-generation sequencing, quality control, and assembly

Viral ribonucleic acid was extracted from 140 μL of swab sample using the QIAamp Viral RNA Mini extraction kit and manufacturer’s guidelines (Qiagen, Hilden, Germany). The viral RNA was reverse transcribed into cDNA and the entire genome amplified using the multi-segment real-time polymerase chain reaction (M-RTPCR) (Zhou et al. 2017), universal IAV Uni/Inf primers IAV Uni/Inf primers, and SuperScript III One-Step RT-PCR with Platinum *Taq* High Fidelity (Invitrogen) at standardised thermocycling conditions (Supplementary methods).

Following M-RTPCR, amplified viral RNA libraries were loaded and sequenced using the Illumina MiSeq platform (Illumina Inc., San Diego, California, USA) at a KEMRI-Wellcome Trust Programme collaborating laboratory in Kilifi (Kenya), as described previously (Nabakooza et al. 2021).

Raw MiSeq sequence reads were de-duplicated and cleaned of any PCR contaminants. The clean reads were assembled using the reference-based FLU module of the Iterative Refinement Meta-Assembler (IRMA) v0.6.7 at a median read quality score (*Q*-score) filter of 30; a minimum read length of 125; frequency threshold for insertion and deletion refinement of 0.25 and 0.6, respectively; a mismatch penalty of 5; and a gap opening penalty of 10 (Shepard et al. 2016).

### 2.3 Secondary sequence data

WG sequences for clade references and vaccine viruses used to classify Uganda virus sequences into genetic clades were downloaded from the Global Initiative on Sharing All Influenza Data (GISAID) EpiFlu database (https://platform.gisaid.org/epi3/cfrontend, accessed on 14 August 2020).

For the continental analysis, WG sequences for Africa H1N1pdm09 and H3N2 virus were downloaded from GISAID (accessed on 14 August 2020). Gene sequences with ambiguous bases ‘Ns’ and 100 nucleotides shorter or longer than the actual gene lengths were excluded. Eligible sequences were aligned per gene per subtype using a codon-aware aligner (https://github.com/veg/hyphy-analyses/tree/master/codon-msa).

### 2.4 Detection of intra-subtype reassortment using Graph Incompatibility Based Reassortment Finder (GiRaF)

We grouped sequences per gene for every subtype into three datasets (D1, D2, and D3). Datasets D1 contained only the newly generated Uganda virus sequences per gene, D1.H1N1pdm09 (*n* = 100) and D1.H3N2 (*n* = 93). Datasets D2 contained both the newly generated Uganda and downloaded Africa viral sequences per gene, D2.H1N1pdm09 (*n* = 758) and D2.H3N2 (*n* = 1224). Datasets D3 (D3.H1N1pdm09 and D3.H3N2) contained the newly generated Uganda, clade references, and vaccine virus sequences per gene. The number of sequences in D3 varied because some clade reference and vaccine viruses lacked all eight gene segments. For the HA gene, D3.H1N1pdm09 had 131 and D3.H3N2 had 132 sequences. Gene sequences in both datasets were aligned per gene per subtype using MUSCLE v3.8.1551 (Edgar 2004).

We ran two simultaneous runs of MrBayes v 3.2.7 (Huelsenbeck and Ronquist 2001) on the Ugandan (D1) and African datasets (D2) for 250 million and 1,000 million generations, respectively, under a GTR + I + Γ substitution model, and sampled trees at every 200,000th generation. The runs converged with an average standard deviation of split frequency < 0.01, tree length estimated sample size (ESS) value > 200,000, tree length potential scale reduction factors (PSRF) of 1.0, and a maximum split frequency PSRF of 1.00–1.002 (Ronquist et al. 2020). The first 25 per cent of trees from each of the two runs were manually discarded (burn-in), and the remaining trees were combined and processed using the GiRaF program (Nagarajan and Kingsford 2011) to detect reassortment at the following settings: burn-in = 0, confidence threshold for reporting a reassortment (threshold= 0 · 70), allowed to test large candidate sets as reassortants and do not consider splits that happen in fewer than *F* fraction of the trees (*F* = 0 · 05).

GiRaF uses a fast biclique enumeration algorithm and statistical tests to search large sets of Markov chain Monte Carlo (MCMC)-sampled phylogenies from different gene segments for incompatible splits. GiRaF infers sets of reassortant viruses if the viruses occupy high-confidence incompatible splits in any of the two gene trees being compared. For a more comprehensive analysis, we set GiRaF to identify reassortment in all twenty-eight pairs of the eight genes. We did 20 repeat runs for both MrBayes and GiRaF per gene for the Uganda datasets (D1 with *n* < 200), but single MrBayes (1,000 M generations) and GiRaF run for the African datasets (*n* > 700) due to computational constraints.

### 2.5 Combining GiRaF results from multiple gene segments

Only reassortment events with a high confidence value (≥0.70) and reassortant sets predicted in at least three of twenty-eight gene pairwise comparisons, as recommended by the GiRaF developer (Nagarajan and Kingsford 2011), and run frequency ≥50 per cent (≥10/20 for Ugandan dataset D1) as previously used in a global H3N2 study (Westgeest, Russell, and Lin 2014) were reported. We confirmed that a given set of reassortant viruses had acquired a new gene if the viruses were reassorted in at least three of twenty-eight gene pairs and if that gene was involved in at least three unique gene pairs. Reassortant sets predicted in more than seven gene pairwise comparisons indicate viruses acquired more than one new gene (Nagarajan and Kingsford 2011). The temporal and spatial distribution of reassortants with different architecture were visualised using ggplot2 in R v4.0.4 (https://www.r-project.org).

### 2.6 Estimating reassortment rates among Africa IAVs

The rate of reassortment (the number of reassortments events per lineage per year) for the Uganda (D1) and Africa datasets (D2) were estimated using a coalescent reassortant constant population model (CoalRe) (Müller et al. 2020) in BEAST2 v.2.6.6 (https://www.beast2.org/) with parameters: GTR + I + G4 substitution model, strict clock, prior for reassortment rate = exponential with mean 0.25, chain length of 500 million, and 10 per cent burn-in.

### 2.7 Phylogenetic clustering and genetic clade classification

Sequence datasets D3 per gene and per subtype were used to generate 36,000 trees using BEAST v 1.10.4 (http://beast.bio.ed.ac.uk) with parameters: GTR + I + G4 substitution model, uncorrelated log-normal relaxed clock, GMRF Bayesian Skyride coalescent prior, UPGMA starting tree, uniform prior for clock rate with an initial value of 0.005, exponential prior for geographical transition rate with a mean of 0.1 migration event per lineage per year, MCMC chain of 100 million, sample frequency of 2,500, and 10 per cent burn-in. All MCMC runs converged with ESS for the tree age, length, and likelihood >800. The maximum clade credibility (MCC) trees were annotated using Tree Annotator v1.10.4 (https://beast.community/treeannotator). Statistical uncertainly was reflected in values of the 95 per cent highest probability density (HPD).

PhyCLIP v2.0 (Han et al. 2019) was used to infer clusters from the HA gene MCC tree per subtype, and the clusters were confirmed as global genetic clades 1–8 (H1N1pdm09) and 1–7 (H3N2) using the European Centre for Disease Prevention and Control (ECDC) reference-based method (ECDC 2010). PhyCLIP uses linear integer programming optimization to assign sequences to a cluster that may share epidemiological linkage, while the ECDC method classifies a cluster of sequences to a genetic clade based on the unique amino acid substitutions in their HA1 or HA2 proteins. Sequences in the remaining seven gene MCC trees per subtype were assigned to clades using the clade-classified HA MCC tree as a reference.

MCC trees were visualised in FigTree v1.4.3 (http://tree.bio.ed.ac.uk/software/figtree/).

### 2.8 Statistical analysis

Correlation and regression analysis to determine the degree of association and effect of the number of viral WGs sampled on the number of reassortants identified by GiRaF were done in R software v4.0.4 (https://www.r-project.org).

### 2.9 Ethics

This study was approved by the Makerere University School of Biomedical Sciences Research and Ethics Committee (SBS-REC) (ref: SBS-577) and Uganda National Council of Science and Technology (UNCST) (ref: HS2519).

## 3. Results

### 3.1 Sample characteristics and whole-genome recovery

We successfully sequenced and assembled 82 · 5 per cent (193/234) WGs for H1N1pdm09 (*n* = 100) and H3N2 (*n* = 93) viruses sampled from Uganda between 2010 and 2018. The generated WGs consisted of the complete coding sequences for all eight genes (Nabakooza et al. 2021).

Four swabs, H1N1pdm09 (*n* = 3) and H3N2 (*n* = 1) of the 193 swabs for which WGs were recovered lacked demographic data. Of the remaining 189 swabs with data, 65 · 6 per cent (124/189) and 34 · 4 per cent (65/189) were sampled from ILI and SARI patients, respectively. Fifty-six percent (106/189) and 43 · 9 per cent (83/189) of the swabs were sampled from male and female patients, respectively. Thirty-two percent (61/189) of the recovered WGs were from patients aged 1 month to < 2 years old, 38 · 6 per cent (73/189) from 2 to < 5 years old, 18 · 5 per cent (35/189) from 5 to < 15 years old, 9 · 5 per cent (18/189) from 15 to < 50 years old, 1 · 1 per cent (2/189) from 50 to < 65 years old, and none from above 65 year olds (Nabakooza et al. 2021).

Seventy-eight percent (151/193) of WGs generated were from swabs sampled from Central (Kampala and Entebbe), 9 · 8 per cent (19/193) from Western (Mbarara and Fort Portal), 7 · 3 per cent (14/193) Northwest (Arua and Koboko), and 4 · 7 per cent (9/193) Eastern Uganda (Tororo) (Fig. 1A). At the continental level, we analysed 785 H1N1pdm09 and 1,224 H3N2 WGs sampled from 38 · 9 per cent (21/54) countries for 12 (2009–20) and 27 (1994–2020) years, respectively, including the newly generated Uganda WGs. The sampled countries spanned Western, Central, Northern, Eastern, Southeast, and Southern Africa (Nabakooza et al. 2021).

### 3.2 Intra-subtype reassortment among influenza A viruses in Uganda

GiRaF predicted intra-subtype reassortment events among H1N1pdm09 and H3N2 viruses involving 3–12 unique gene pairs with high confidence (0 · 99–1). The GiRaF confidence measures the degree of incompatibility of one or more viral sequences in the phylogeny. We reported only reassortment events and reassortant sets predicted in ≥50 per cent of the 20-repeat GiRaF runs, as used previously (Westgeest, Russell, and Lin 2014), and reassorted in ≥3 unique gene pairs (Nagarajan and Kingsford 2011).

**Figure 2.**
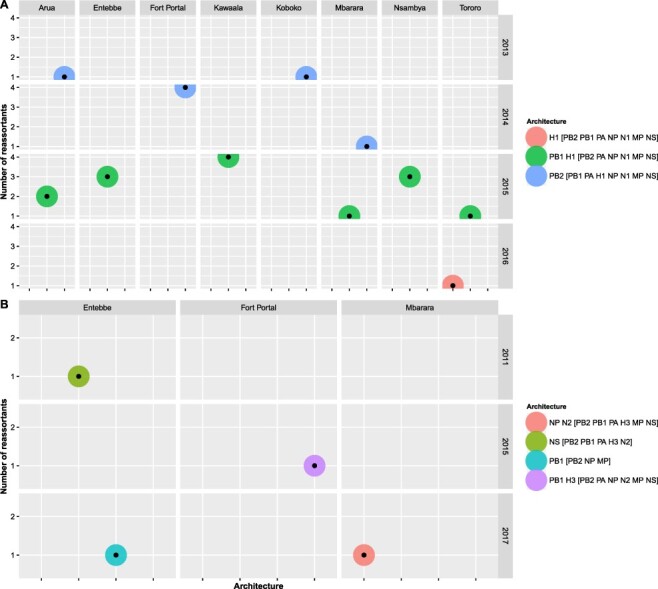
Temporal and spatial distribution of reassortant influenza A viruses with different reassortment architecture in Uganda. Panel A shows the distribution of reassortants with different reassortment architecture among 100 Uganda H1N1pdm09 viruses sampled in 2010–18. Panel B shows the distribution of reassortants with different reassortment architecture observed among 93 Uganda H3N2 viruses sampled in 2010–17. Reassortants and the genes involved were identified using GiRaF software (Nagarajan and Kingsford 2011). Details on the number of Uganda H1N1pdm09 and H3N2 reassortants with a specific architecture per site per year are provided in Supplementary Tables S3 and S4, respectively.

GiRaF identified 19 reassortment events and 3 reassortant sets containing 22 unique reassortants among the 100 newly generated Uganda H1N1pdm09 viruses (Supplementary Table S1). The H1N1pdm09 reassortants circulated between August 2013 and February 2016. Sixty-three percent (14/22) of the reassortants, sampled in June–November 2015, acquired both new PB1 and H1 genes. In total, 31 per cent (7/22) and 4 · 5 per cent (1/22) of reassortants acquired new PB2 and H1 genes, respectively, and were sampled in the 2013–14 and 2016 seasons, respectively (Fig. 2A).

There were thirty-one reassortment events and four single-taxa reassortant sets predicted among the ninety-three newly generated Uganda H3N2 viruses (Supplementary Table S2). The first H3N2 reassortant (EBB2779) sampled in Entebbe on 29 September 2011 had a new non-structural (NS) gene. Another reassortant (FTL1393) sampled in Fort Portal on 8 December 2015 had new H3 and PB1 genes. MBA1094 with new NP and N2 and EBB6461 with a new PB1 gene were sampled on 14 April and 15 June 2017, respectively (Fig. 2B).

We observed more reassortment events but smaller reassortant sets among Uganda H3N2 than H1N1pdm09 viruses. There was no clear geographical pattern in the distribution of H1N1pdm09 reassortants, while the H3N2 reassortants were observed sporadically in only Central and Western Uganda (Fig. 2).

The coalescent-based analysis (Müller et al. 2020) estimated the Uganda H1N1pdm09 and H3N2 viruses evolved at mean rates of 2 · 76 × 10^−3^ [95 per cent HPD, 2 · 54–2 · 99 × 10^−3^] and 2 · 73 × 10^−4^ [95 per cent HPD, 1 · 938–3 · 53 × 10^−4^] mutations per site per year across all eight genes and reassorted at mean rates of 0.2668 [95 per cent HPD, 0.1237–0.4255] and 0.0216 [95 per cent HPD, 0 · 00912–0.0355] events per lineage per year, respectively.

### 3.3 Genetic clade classification and clade switching among Uganda influenza A viruses

Both Uganda H1N1pdm09 and H3N2 viruses showed varying phylogenetic patterns across all the eight genes and were classified into genetic clades based on the characteristic substitutions in the HA1 of their antigenic H1 and H3 proteins, respectively. Knowing the clades in which circulating viruses belong is key in detecting new antigenic variants for effective vaccine virus selection.

**Figure 3.**
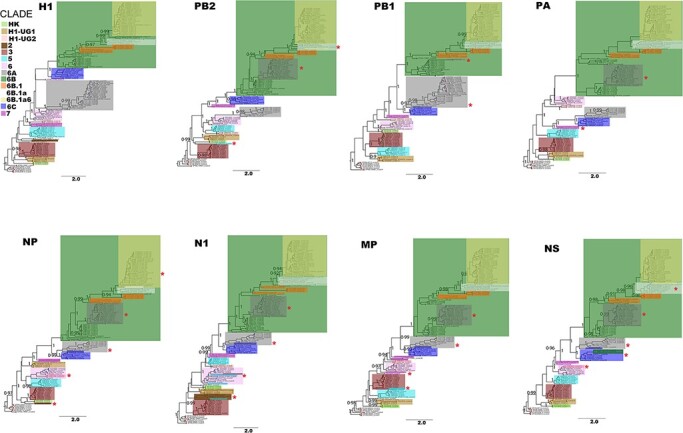
Genetic clade classification and clade switching across whole genomes of 100 H1N1pdm09 viruses sampled between 2010 and 2018 in Uganda. Viruses were classified into genetic clades based on unique amino acid substitutions in the HA1 subunit of the H1 protein(ECDC 2010). Clades in the remaining seven genes (PB2, PB1, PA, NP, N1, MP, and NS) were inferred using the H1 classified phylogeny as a reference. Original clade colours (based on the H1 phylogeny) of viruses that switched clades were maintained for visualisation purposes. Altered clades with additional virus(es) from the original clade are marked with (*). Sample nodes are coloured based on their PhyCLIP cluster ID.

PhyCLIP grouped Uganda H1 sequences into five clusters belonging to five ECDC global genetic clades: A/Hong Kong/2213/2010 (HK), 3, 5, 6, and 7, and two novel clades H1-UG1 with D222E and I267T, and H1-UG2 with N97D and V321I that circulated in 2010–11 (Fig. 3).

Characteristic HA1 substitutions among clade 3 viruses were A134T and S183P, clade 5 (D97N, R205K, I216V, and V249L), clade 6 (D97N, S185T, and S203T), clade 7 (S143G, S185T, and A197T), and HK (V19I, N97D, and S128P). Clade 6 viruses diverged into 6A, 6B, and 6C, with subclades of 6B dominating through 2018.

**Figure 4.**
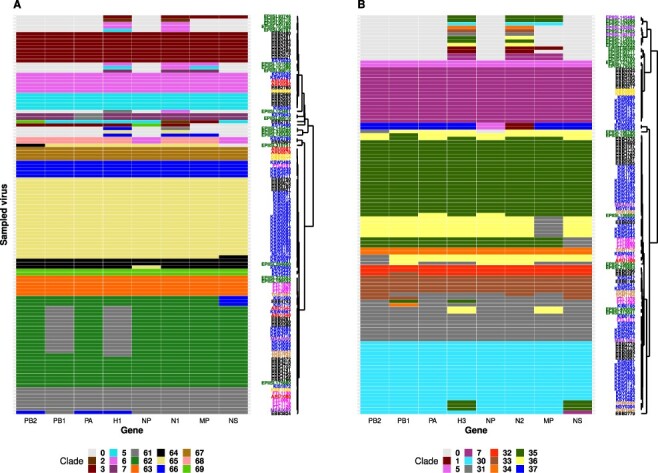
Genomic patterns resulting from clade switching across the whole-genome of Influenza A viruses sampled between 2010 and 2018 in Uganda. Panel A: Genomic patterns observed among 100 H1N1pdm09 viruses. Numeric **0** = gene segment not available, **2** = clade 2, **3** = clade 3, **5** = clade 5, **6** = clade 6, **7** = clade 7, **61** = clade 6A, **62** = clade 6B, **63** = clade 6B.1, **64** = clade 6B.1a, **65** = Clade 6B.1a6, **66** = Clade 6C, **67** = novel clade H1-UG1, **68** = novel clade H1-UG2, **69** = A/Hong Kong/ 2213/2010 clade (HK). Panel B: Genomic patterns observed among 93 Uganda H3N2 viruses. Numeric 0 = gene segment not available**, 1** = clade 1, **5** = clade 5, **7** = clade 7, 30 = clade 3B, **31** = clade 3C.2a, **32** = clade 3C.2a1a, **33** = clade 3C.2a1b, **34** = clade 3C.2a3, **35** = clade **3C.3**, **36** = clade 3C.3a, and **37** = novel clade H3-UG1. Virus sample codes are coloured based on sampling site: Arua-**ARU**, Entebbe-**EBB**, Fort Portal-**FTL**, Koboko-**KBK**, Mbarara-**MBA**, Tororo-**TOR**, Kampala (Kibuli-**KIB**, Nsambya-**NSY**, Kawaala & Kiswa-**KSW**, Kisenyi -**KSY**, and Kitebi -**KIS**), other African country-**EPIISL**, and global country-**EPIISL.**

Twenty-four percent of the newly generated H1N1pdm09 viruses switched clades in at least one of the remaining seven genes (PB2, PB1, PA, NP, N1, MP, and NS). Sixty-two percent (15/24) of H1N1pdm09 viruses had a clade 6A-like H1 and 6B-like PB2, PA, NP, N1, MP, and NS genes (Fig. 4A). Other H1N1pdm09 viruses switched from clade 3 to HK (*n* = 1), H1-UG2 to clade 6 (*n* = 2), 6B to 6C (*n* = 3), 6B.1a to 6B.a16 (*n* = 1), 6B.1a6 to 6B.1a (*n* = 1), and one clade 5 virus (EBB2707) had an HK-like PB2, clade 2-like N1, and clade 3-like MP gene.

**Figure 5.**
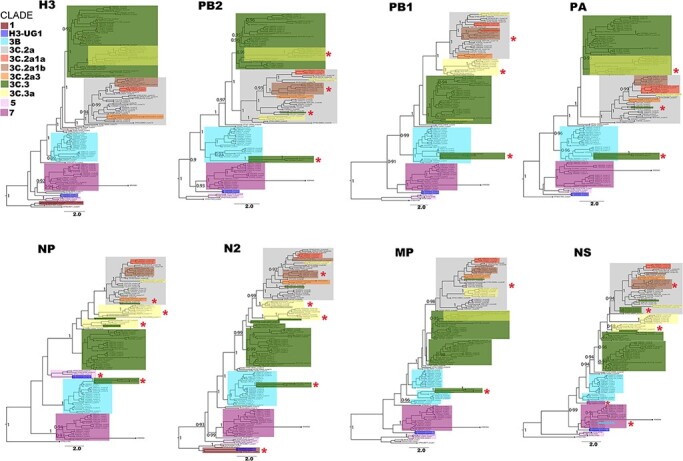
Genetic clade classification and clade switching across whole genomes of 93 H3N2 viruses sampled in 2010–18 in Uganda. Genetic clades and colour codes, markings were determined and used as in Fig. 3 above. Sample nodes are coloured based on their PhyCLIP cluster ID.

PhyCLIP grouped the ninety-three Uganda H3 sequences in eight clusters belonging to two ECDC global genetic clades 3 and 7 and a novel clade H3-UG1 (Fig. 5). Clade 3 viruses had HA1 substitutions A198S, V223I, and N312S, clade 7 had S45N, K62E, K144N, T212A, and S214I, and the two H3-UG1 viruses sampled in 2010 had N45S and P289S. Clade 3 diverged into clades 3B, 3C.2, and 3C.3 and dominated through 2017.

Twenty-three percent (22/93) of the newly generated H3N2 viruses switched clades in at least one of the remaining 7 genes (Fig. 4B). Sixty-eight percent (15/22) of the viruses switched from clade 3C (3C.3 or 3C.3a) to clades 3B or 3C.2a in the PB2, PB1, PA, NP, N2, MP, and NS genes. Other viruses switched from 3B to 7 (*n* = 1), 3C.2a to 3C.2a1b or 3C.2a3 (*n* = 3), 3C.2a1a to 3C.2a1b (*n* = 1), and the two H3-UG1 viruses (KSW0643 and KSW0659) had clade 5-like NP and clade 1-like N2 genes.

### 3.4 Intra-subtype reassortment among Africa influenza A viruses

GiRaF predicted reassortment events and reassortants among Africa H1N1pdm09 and H3N2 viruses with high probability confidence values of 0 · 94–1 and 0 · 99–1, respectively.

### 3.5 Africa H1N1pdm09 viruses

Reassortment events occurred at a frequency of 12 · 4 per cent (94/758) among the 758 Africa H1N1pdm09 viruses. GiRaF identified 18 reassortant sets containing 13 · 3 per cent (101) unique reassortants among the 758 Africa H1N1pdm09 viruses. Sixty-one percent (11/18) of the reassortant sets contained 2–74 viruses and the remaining seven sets had a single virus (Supplementary Table S5).

**Figure 6.**
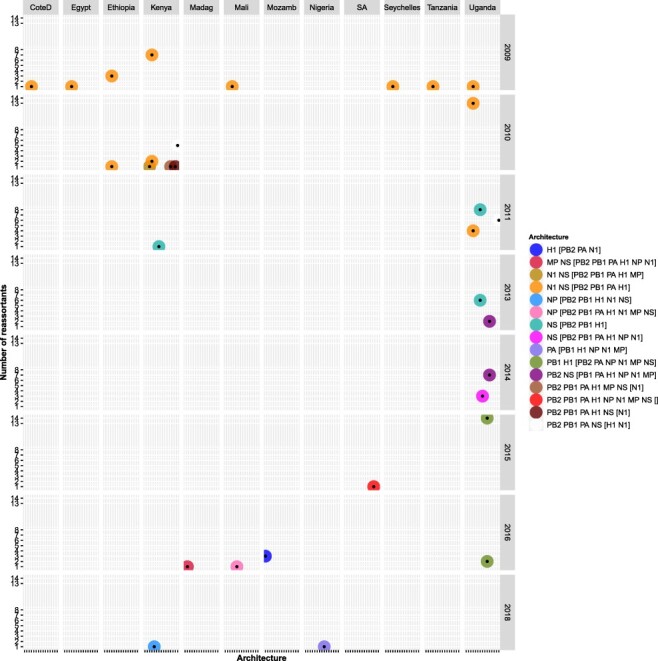
Temporal and spatial distribution of H1N1pdm09 reassortant viruses with different reassortment architecture among the 758 H1N1pdm09 viruses sampled between 2009 and 2020 in Africa. Long country names were abbreviated for visualisation purposes as: CoteD for CoteD’ivoire, Madag for Madagascar, Mozamb for Mozambique, and SA for South Africa. Reassortants and the genes involved were identified using GiRaF software (Nagarajan and Kingsford 2011). Details on the number of Africa H1N1pdm09 reassortants with a specific architecture per country per year are provided in Supplementary Table S3.

Using the African WG dataset (D2), GiRaF identified additional forty viruses from the newly generated Uganda dataset and four previously sampled Uganda viruses [2009 (*n* = 1), 2014 (*n* = 2), 2016 (*n* = 1)] had reassorted. The first Uganda H1N1pdm09 reassortant, EPIISL62338, was sampled on 18 August 2009, reassorted in six unique gene pairs, and had new N1 and NS genes (Fig. 6).

**Figure 7.**
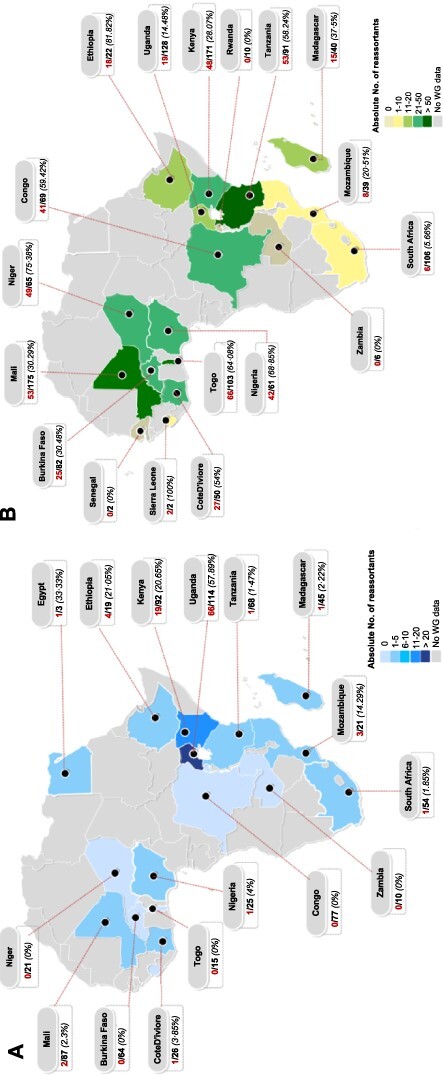
Spatial distribution of influenza A virus reassortants across Africa. The numerator (red) is the absolute number of reassortants and the denominator (black) is the number of whole genomes sampled or analysed from a given country. Panel A shows the distribution of reassortant H1N1pdm09 viruses sampled between 2009 and 2020 in Africa. The number of H1N1pdm09 reassortants in Seychelles (*n* = 1) were not shown. Panel B shows the distribution of reassortant H3N2 viruses sampled between 1994 and 2020 in Africa. The frequency of reassortment per country is shown in (%). Countries are colour-coded based on the absolute number of reassortant viruses identified by GiRaF (Nagarajan and Kingsford 2011).

At the continental level, reassortants were observed in 63 · 2 per cent (12/19) of countries with H1N1pdm09 virus WGs sampled (Fig. 7A). Thirty-six percent (37/101) of Africa H1N1pdm09 reassortants had both new N1 and NS (architecture: N1 NS [PB2 PB1 PA H1] or N1 NS [PB2 PB1 PA H1 MP]), and were sampled in Eastern, Western, and Northern regions (Fig. 6). Uganda had the highest frequency of reassortants 57 · 9 per cent (66/114), contributing 65 · 3 per cent (66/101) to Africa’s H1N1pdm09 reassortant pool. This was followed by Kenya 18 · 8 per cent (19/101), Ethiopia 3 · 96 per cent (4/101), Mozambique 2 · 97 per cent (3/101), and Mali 1 · 98 per cent (2/101), while the remaining countries contributed 0–0 · 99 per cent. H1N1pdm09 reassortants were widespread in the Eastern 91 · 1 per cent (92/101), Western (4/101), Southeast (3/101), Northern (1/101), and Southern (1/101), but none in Central Africa (Fig. 7A).

**Figure 8.**
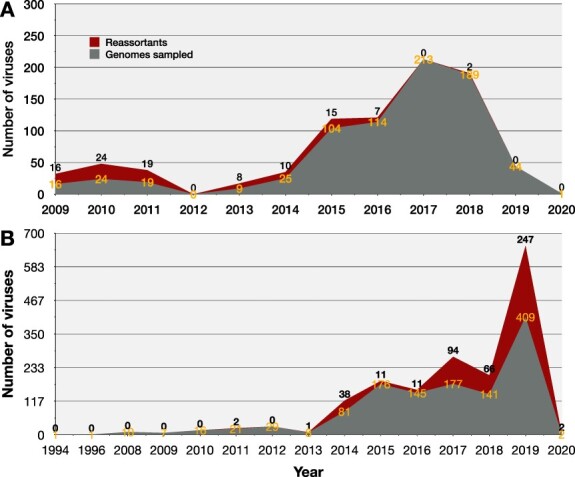
Temporal distribution of influenza A virus reassortants across Africa. Temporal distribution of influenza A virus reassortants in Africa. Panel A shows the distribution of H1N1pdm09 whole genomes and reassortants sampled in 2009 through 2020 in Africa. Panel B shows the distribution of H3N2 whole genomes and reassortants sampled between 1994 and 2020 in Africa.

All Africa H1N1pdm09 viruses sampled in 2009–11 were identified as reassortants by GiRaF, peaking in 2010 (Fig. 8A). The earliest reassortant (EPIISL34539) was sampled on 16 June 2009 in CoteD’ivoire and had new N1 and NS genes. No WGs were sampled in 2012. The number of reassortants increased from 8 to 15 between 2013 and 2015 and drastically declined between 2017 and 2019 despite increased WG sampling. None of the H1N1pdm09 viruses sampled in 2020 (as of 3rd January) were identified as reassorted. The latest reassortant (EPIISL353346) had a new PA gene and was sampled on 7 November 2018 in Nigeria. Viral reassortants with similar architecture persisted in circulation for 1–3 consecutive years in Africa (Fig. 6).

The coalescent model analysis estimated a mean evolutionary rate of 2 · 7 × 10^−3^ [95 per cent HPD, 2 · 599–2 · 801 × 10^−3^] mutations per site per year across all eight genes, and a mean reassortment rate of 0.1788 [95 per cent HPD, 0.1227–0.2366] events per lineage per year among Africa H1N1pdm09 viruses.

### 3.6 Africa H3N2 viruses

We observed 256 (20 · 9 per cent) reassortment events and 46 reassortant sets containing 472 (38 · 6 per cent) unique reassortants among the 1,224 Africa H3N2 viruses. Of the reassortant sets, 18 and 28 had a single and 2–192 viruses, respectively (Supplementary Table S6).

**Figure 9.**
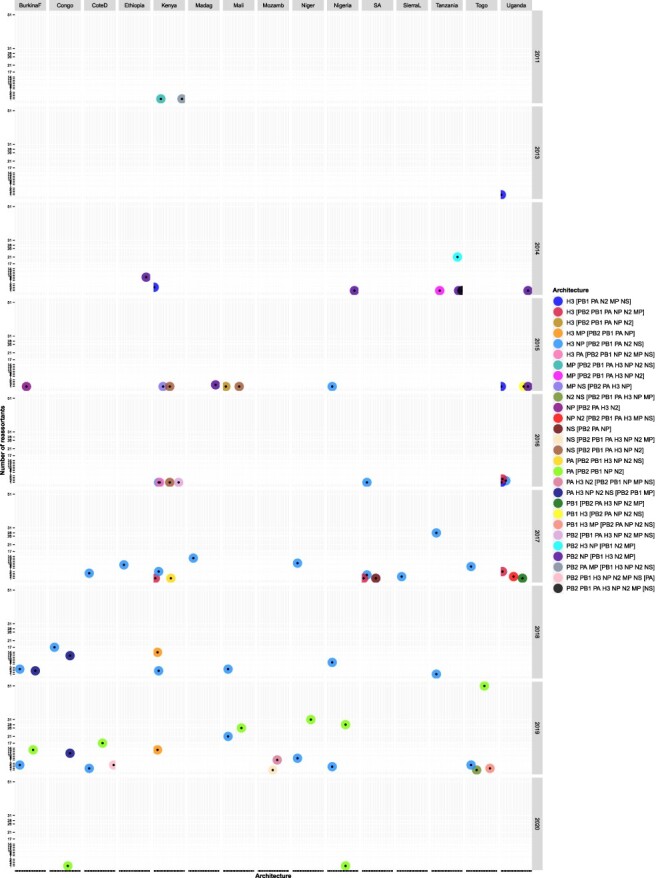
Temporal and spatial distribution of H3N2 reassortant viruses with different reassortment architecture among the 1224 H3N2 viruses sampled in 1994–2020 in Africa. Reassortants and the genes involved were identified using GiRaF software (Nagarajan and Kingsford 2011). Long country names were abbreviated for visualisation purposes as: BurkinaF for Burkina Faso, CoteD for CoteD’ivoire, Madag for Madagascar, Mozamb for Mozambique, SA for South Africa, SierraL for Sierra Leone. Details on the number of Africa H3N2 reassortants with a specific architecture per country per year are provided in Supplementary Table S4.

Using the African H3N2 WG dataset (D2), GiRaF identified the earliest H3N2 reassortant in Uganda as TOR0492 with a new H3 gene, sampled on 13 January 2013 (Fig. 9). Notably, GiRaF identified additional sixteen viruses from the newly generated Uganda H3N2 dataset as reassortants at the African level. However, one (EBB2779) of the four reassortants identified in the Ugandan (D1) was not a reassortant in the African dataset (D2).

The earliest Africa H3N2 reassortant, EPIISL394106, had new PB2, PA, and MP genes and was sampled on 20 October 2011 in Kilifi (Kenya). Since the first two reassortants (EPIISL394106 and EPIISL393689) were sampled from Kilifi in October–November 2011, H3N2 reassortants were next sampled in 2013 in Uganda (Fig. 9). Strikingly, the number of reassortants increased drastically to 38 in 2014 and plateaued to 11 in 2015–16 (Fig. 8B). There was a sharp increase to 94, 66, and 247 reassortants in 2017, 2018, and 2019, respectively. Thirty-five percent (166/472) of H3N2 reassortants sampled across Africa (except Northern region) between 2015 and 2019 had new H3 and NP genes (architecture, H3 NP [PB2 PB1 PA N2 NS]). Also, 35 · 6 per cent (168/472) sampled from Western and Central Africa between 2019 and 2020 had new PA (architecture, PA [PB2 PB1 NP N2]) (Fig. 9 and Supplementary Table S4).

Togo had the highest absolute number of H3N2 reassortants 64 · 1 per cent (66/103), followed by Mali 30 · 3 per cent (53/175) and Tanzania 58 · 2 per cent (53/91) (Fig. 7B), contributing 13 · 98 per cent (66/472), 11 · 2 per cent (53/472), and 11 · 2 per cent (53/472) to the Africa H3N2 reassortants pool, respectively. The Western contributed majorly 55 · 9 per cent (264/472) to the reassortant pool than Eastern 32 · 42 per cent (153/472), Central 8 · 7 per cent (41/472), Southeast 1 · 7 per cent (8/472), Southern Africa 1 · 3 per cent (6/472), and none in Northern Africa. Africa H3N2 reassortants with similar architecture persisted in circulation for 2–5 consecutive years at both country (Congo, South Africa, Kenya, and Uganda) and region (Eastern and Western) levels. All persistent reassortants had new H3 genes (Fig. 9 and Supplementary Table S4).

### 3.7 Association between the number of sampled genomes and reassortants observed

There was a moderate and strong positive correlation between the number of WGs sampled and reassortants observed among Africa H1N1pdm09 (Spearman’s rank correlation rho = 0 · 415, *P*-value = 0.077; adjusted *R-*squared = 0.3328, *P*-value = 0.0057) and H3N2 viruses (Spearman’s rank correlation rho = 0 · 76, *P*-value = 0.0001; adjusted *R*-squared = 0.4555, *P*-value: 0.0009), respectively (Supplementary Figs. S7 and S8).

## 4. Discussion

We successfully recovered 82 · 5 per cent (193/234) viral WGs directly from frozen patients’ swabs sampled in Uganda from 2010 to 2018, which is comparable to WG recovery rates of 82–88 per cent previously reported in developed countries (Zhou et al. 2017; Simon et al. 2019). This is proof that African surveillance laboratories are equally efficient in sample collection, processing, and storage to support next-generation genomic sequencing and analyses.

Here, we inferred a reassortant as a virus containing one or more genes originating from more than one parent virus of the same subtype co-infecting a host cell. However, parent viruses could be of different subtypes (Schrauwen et al. 2011; Phipps et al. 2017).

WG analysis revealed a higher frequency of reassortment events and reassortants among Africa H3N2 [20 · 9 per cent (256/1,224) and 38 · 6 per cent (472/1,224)] than among H1N1pdm09 viruses [12 · 4 per cent (94/758) and 13 · 3 per cent (101/758)], respectively. These observed differences highlight differences in drivers of gene exchanges within subtypes. However, the detection of reassortants depends on the number and uniqueness (genetic diversity) of the viruses sampled per subtype (Maljkovic Berry et al. 2016; Villa and Lässig 2017). Reassorted IAVs observed in Uganda clustered with earlier viruses sampled globally, indicative of intercontinental importations of reassortants to Africa. Viral reassortants with a specific architecture circulated in Africa for up to five consecutive years at country and region levels.

Uganda–Africa comparisons showed that reassortment events were more frequent among Uganda H1N1pdm09 and H3N2 at 0 · 19 and 0 · 33, respectively, than Africa H1N1pdm09 (0 · 124) and H3N2 (0 · 209) viruses, as reported previously (Maljkovic Berry et al. 2016). Africa H3N2 underwent more intra-subtype reassortment events involving larger reassortant sets than H1N1pdm09 viruses, consistent with global results. However, the overall event frequencies were higher among Africa IAVs than 0.0335–0.0345 estimated among 1,188 global H3N2 viruses (Maljkovic Berry et al. 2016). GiRaF’s reassortant frequencies among Uganda H1N1pdm09 (0.22), and Africa H1N1pdm09 (0 · 133) and H3N2 viruses (0 · 3856) were comparable to Maljkovic’s range of 0.139–0.681 (Maljkovic Berry et al. 2016). GiRaF identified more newly generated Uganda viruses as reassortants at the African than Ugandan level. Thus, our results show a positive correlation between the number of genomes sampled and the proportion of reassortants observed.

Interestingly, our GiRaF-based event frequencies were comparable with the coalescent-based reassortment rates among the Ugandan 0.2668 (95 per cent HPD, 0.1237–0.4255) and African H1N1pdm09 viruses 0.1788 (95 per cent HPD, 0.1227–0.2366 events/lineage/year). Overall, reassortment rates among Africa H1N1pdm09 viruses (0.1237–0.4255) were comparable to the 0 · 15–0 · 8 events/lineage/year estimated among global pandemic H1N1pdm09 viruses (Müller et al. 2020). Shockingly, Uganda H3N2 viruses reassorted at a lower mean rate of 0.0216 (95 per cent HPD, 0 · 00912–0.0355) than the global estimate of 0 · 35–0 · 65 events/lineage/year (Müller et al. 2020). Although H3N2 viruses undergo frequent reassortment events globally, the low rate estimated among Uganda H3N2 viruses might be due to the resulting reassortants being unfit and negatively selected hence are not detected in appreciable frequencies (Neverov et al. 2014; McDonald et al. 2016; Villa and Lässig 2017; Potter et al. 2019). Furthermore, some unfit reassortants could have gone extinct before sampling due to the limited and inconsistent geographical and temporal viral sampling in the Uganda surveillance caused by fluctuating foreign funds. Unfortunately, we did not report the reassortment rate for the larger Africa H3N2 dataset (*n* = 1,224) due to computational difficulties obtaining MCMC convergence.

GiRaF predicted larger reassortant sets among Africa H3N2 than H1N1pdm09 viruses, indicative of increased transmissibility of the H3N2 reassortants. In total, 78 · 2 per cent (79/101) of Africa H1N1pdm09 acquired new NS genes that highlight the importance of the NS protein in suppressing the host’s innate immunity, increasing viral replication, transmissibility, virulence, pathogenicity, and pandemic potential as well as host range (Hao, Wang, and Li 2020; Rosário-Ferreira et al. 2020). Fifty-seven percent (273/472), 50 per cent (236/472), and 43 · 43 per cent (205/472) of Africa H3N2 viruses reassorted in the H3, NP, and PA genes, respectively. The high instability of H3 genes among Africa H3N2 viruses could be driven by immunoselection, as reported previously (Lubeck, Palese, and Schulman 1979; Rabadan, Levine, and Krasnitz 2008).

Uganda IAVs formed varying phylogenetic patterns across the genome indicative of reassortment within subtypes. PhyCLIP grouped sequences into clusters corresponding to the global genetic clades but lacked the resolution to identify new emerging subclades with minor genetic changes. Thus, statistical-based clustering should be used with caution or complemented with genetic-based analysis for influenza virus classification. Manual comparisons of gene trees identified 24 H1N1pdm09 and 22 H3N2 viruses that switched clades in at least one of PB2, PB1, PA, NP, NA, MP, and NS genes. However, GiRaF did not predict 8 · 33 per cent (2/24) H1N1pdm09 and 45 · 45 per cent (12/22) H3N2 virus clade switchers as reassortants. This is expected because clades are determined based on unique substitutions resulting from mutations rather than gene exchange. Similarly, 16 · 7 per cent (1/6) and 50 per cent (4/8) of Uganda H1N1pdm09 and H3N2 viruses that formed long branches, respectively, were not reassortants. Long branches reflect an increase in viral genetic divergence due to accumulated amino acid substitutions (Westgeest, Russell, and Lin 2014) rather than reassortment.

Our study utilised over 2,000 WGs and confirmed the previous speculation of reassortment among Africa IAVs (Nelson et al. 2014). The earliest H1N1pdm09 (EPIISL34539) and H3N2 (EPIISL394106) reassortants were sampled on 16 June 2009 and 20 October 2011 in CoteD’ivoire and Kilifi (Kenya), respectively. Contrary to a previous Kenyan study (Gachara et al. 2016) that used the FluGenome tool (Lu et al. 2007), GiRaF identified eight H1N1pdm09 viruses (EPIISL140406- EPIISL140413) sampled from Kenya in January–June 2010 as reassortants. A global GiRaF analysis detected three H3N2 reassortants (EPIISL127589, EPIISL111134, EPIISL111314) sampled in Johannesburg (South Africa) between 1994 and 1997 (Westgeest, Russell, and Lin 2014). Our GiRaF analysis also predicted the A/Johannesburg/33/1994 virus (EPIISL127589), with all eight genes, as a reassortant in nine events (mostly in the H3 gene) involving eight different reassortant sets with high confidence 0 · 99–1. However, the sets were predicted in only 1–2 unique gene pairs hence were not reported. This observation highlights the specificity of GiRaF in detecting reassortment regardless of the geographical location. However, the number of reassortants observed depends on the number of genomes analysed per location.

Eastern and Western Africa formed the hubs for H1N1pdm09 and H3N2 reassortants in Africa, respectively. However, we used pre-collected data with a biased sampling between districts (sites) and countries. For example, there were no IAV WGs sampled in 2012 in Africa. Due to financial limitations, we sequenced 24 · 1 per cent (234/971) of the available viral swabs from Uganda, 8 · 1 per cent (19/234) of which failed quality control and next-generation sequencing (NGS). Therefore, our current results show an uneven geographical distribution of reassortants, and the reassortant frequencies may change as under-sampled districts and countries generate more IAV WGs.

Future studies should integrate genomic data from different species and geographical regions to investigate whether the reassorted human IAVs originate from within through zoonotic exchanges, persistence, and local genomic mixing or outside Africa (Nelson et al. 2014; Meseko et al. 2015; Owuor 2021). Furthermore, studies should investigate inter-subtype reassortment among co-circulating viruses (Schrauwen et al. 2011), adopt Bayesian analysis to estimate the time of reassortment events, and phenotypic analysis to assess the antigenic novelty of observed influenza reassortants.

In conclusion, our study highlights the advantage of analysing whole-over partial genomes in influenza investigations, especially those that influence public health decision-making. The current live-attenuated vaccine production targets only the surface HA and NA genes (Nogales and Martínez-Sobrido 2016). However, our WG analysis shows that IAVs previously circulating in Africa acquired new HA or NA or internal genes. This calls for vaccine developers to strongly consider including other highly reassorting genes like PB2, PB1, NP, and NS in the WHO vaccine production workflow. Furthermore, African influenza surveillance programmes should adopt and implement routine WG and genomic analysis to monitor circulating and detect emerging viruses to inform vaccine selection.

## Supplementary Material

veac005_Supp

## Data Availability

The newly generated Uganda H1N1pdm09 and H3N2 sequences were deposited in the Global Initiative on Sharing All Influenza Data (GISAID) EpiFlu™ database under the accessions EPIISL498819-EPIISL498931 and EPIISL498934-EPIISL499037, respectively. Sequences currently not public. Xml files for CoalRe analysis are publicly available.
