# Supplementary material for: Whole-genome analysis to determine the rate and patterns of intra-subtype reassortment among influenza type-A viruses in Africa
Source: Virus Evol. 2022 Jan 29;8(1):veac005. doi: 10.1093/ve/veac005 (PMC8933723; doi:10.1093/ve/veac005)
Supplement: veac005_Supp [file veac005_supp.zip › VEVOLU-2021-129_Supplementary_Material_Accepted.pdf]

Table of Contents

Supplementary materials and methods 2
Source of swabs and sampling 2
Viral RNA isolation and amplification 2
Next-generation sequencing 2
Sequence quality control 3
Genome assembly using Iterative Refinement Meta-Assembler (IRMA) 3
Viral sequence clustering using the Phylogenetic Clustering by Linear Integer Programming (PhyCLIP) .. 3
Supplementary results 5
Supplementary Table S1: Intra-subtype reassortment among Uganda H1N1pdm09 viruses 5
Supplementary Table S2: Intra-subtype reassortment among Uganda H3N2 viruses 6
Supplementary Table S3: Details on the distribution of H1N1pdm09 reassortant viruses with a specific architecture in Uganda and Africa between 2009 and 2020 7
Supplementary Table S4: Details on the distribution of H3N2 reassortant viruses with a specific architecture in Uganda and Africa between 1994 and 2020 8
Supplementary Table S5: Intra-subtype reassortment events and reassortants among Africa H1N1pdm09 viruses 10
Supplementary Table S6: Intra-subtype reassortment events and reassortants among Africa H3N2 viruses 12
Supplementary Fig. S7: Variation in the number of H1N1pdm09 reassortants and whole genomes sampled 15
Supplementary Fig. S8: Variation in the number of H3N2 reassortants and whole genomes sampled 16

Supplementary materials and methods

Source of swabs and sampling

Nasal and oropharyngeal swabs were collected from outpatients and inpatients with influenza-like illnesses (ILI) and severe acute respiratory illnesses (SARI) at the different sentinel sites, respectively, as described previously (Lutwama et al., 2012). The swabs were tested for influenza (A and B) and the IAV were further subtyped for seasonal [A(H1N1) and A(H3N2)] and pandemic A(H1N1)pdm09 influenza using the Centers for Disease Control's (CDC) real-time reverse-transcription polymerase chain reaction (rRT-PCR) protocols and primers (Atlanta, Georgia)(C.D.C., 2009) (Lutwama et al., 2012) . All patient swabs were uniquely coded and frozen at -80^{0}C, and their sociodemographic data recorded using EpiInfo (CDC, Atlanta) (Lutwama et al., 2012).

Viral RNA isolation and amplification

Following isolation, the viral RNA was reverse transcribed into cDNA and the entire IAV genome amplified using the multi-segment real-time polymerase chain reaction (M-RTPCR) (Zhou et al., 2017) and universal IAV Uni/Inf primers in 25 µL reactions containing 8 µL nuclease-free water, 12.5 µL 2× RT-PCR buffer, 0.2 µL Uni12/Inf1 (10 µM), 0.3 µL Uni12/Inf3 (10 µM), 0.5 µL Uni13/Inf1 (10 µM), 0.5 µL SuperScript III One-Step RT-PCR with Platinum *Taq* High Fidelity (Invitrogen) and 3 µL extracted RNA. The M-RTPCR standardised thermocycling conditions were as follows: 42°C for 50 minutes, 50°C for 10 minutes, 94°C for 2 minutes; 4 cycles (94°C for 30 seconds, 43°C for 30 seconds and 68°C for 3 minutes and 50 seconds) followed by 30 cycles of 94°C for 30 seconds, 57 °C for 30 seconds and 68°C for 3 minutes and 30 seconds (with the 3 minutes and 30 seconds for the 68°C extension step increased by 10 seconds per subsequent cycle after cycle 1); and a final extension step at 68°C for 10 minutes.

Next-generation sequencing

Following PCR, the amplicons were purified using 1X AMPure XP beads (Beckman Coulter Inc., Brea, CA, USA), quantified with Quant-iT dsDNA High Sensitivity Assay (Invitrogen, Carlsbad, CA, USA), and normalized to 0.2 ng/µL. Indexed paired end libraries were then generated from 2.5 µL of 0.2 ng/µL amplicon pool using Nextera XT Sample Preparation Kit (Illumina, San Diego, CA, USA) following the manufacturer's protocol. Amplified libraries were purified using 0.8X AMPure XP beads, quantitated with Quant-iT dsDNA

High Sensitivity Assay (Invitrogen, Carlsbad, CA, USA), and evaluated for fragment size in the Agilent 2100 BioAnalyzer System using the Agilent High Sensitivity DNA Kit (Agilent Technologies, Santa Clara, CA, USA). Libraries were then diluted to 2nM in preparation for pooling and denaturation for running on the Illumina MiSeq (Illumina, San Diego, CA, USA). Pooled libraries were sodium hydroxide denatured, diluted to 12.5 pM and sequenced on the Illumina MiSeq using 2 x 250 bp paired end reads with the MiSeq v2 500 cycle kit (Illumina, San Diego, CA, USA). Five percent Phi-X (Illumina, San Diego, CA, USA) spike-in was added to the libraries to increase library diversity by creating a more diverse set of library clusters. The MiSeq generated paired reads as fastq.gz files for each sample.

Sequence quality control

Raw MiSeq reads were de-duplicated using FastUniq v1.1 (Xu et al., 2012) and Trimmomatic v0·39 (Bolger et al., 2014) was used to trim off Nextera transposase, adaptors, and PCR primers from the unique reads, retaining only reads with ≥80 bps. Clean reads were used as input for the Iterative Refinement Meta-Assembler (IRMA) assembly.

Genome assembly using Iterative Refinement Meta-Assembler (IRMA)

IRMA default settings for IAV genome assembly were as follows: median read quality score (Q-score) filter of 30; minimum read length of 125; frequency threshold for insertion and deletion refinement of 0.25 and 0.6, respectively; mismatch penalty of 5; and gap opening penalty of 10 (Shepard et al., 2016). The IRMA output included: consensus sequences for all the eight gene segments, paired read counts, coverage depth, allele frequencies, and statistically supported variants for each sample.

Viral sequence clustering using the Phylogenetic Clustering by Linear Integer Programming (PhyCLIP)

Maximum likelihood trees were rooted using the oldest sequence A/California/04/2009 for subtype H1N1pdm09 and A/Perth/16/2009 for subtype H3N2. Rooted trees were used as input for PhyCLIP (Han et al., 2019). PhyCLIP clustering was optimised using a series of parameter sets for the minimum number of sequences [*S*, 3-10(1)], false discovery rate (*FDR*, 0.05-0.20(0.05)], multiple of deviations [*gamma*, 1-3(0.5)]

and zero-branch length collapsed. The optimal clustered trees for H1N1pdm09 virus H1 genes were found at parameter S=6, FDR=0·1, gamma=3 , PB2 (S=3, FDR=0·05, gamma=3), PB1 (S=8 FDR=0·2, gamma=2·5), PA (S=3, FDR=0·15, gamma=3), NP (S=4, FDR=0·15, gamma=3), N1 (S=3, FDR=0·15, gamma=2·5), MP (S=3, FDR=0·1, gamma=3), and NS (S=3, FDR=0·1, gamma=2.5). The H3N2 virus H3 gene sequences were optimally clustered at S=4, FDR=0·2, gamma=3, PB2 (S=3, FDR=0·1, gamma=3), PB1 (S=3, FDR=0·05, gamma=3), PA (S=4, FDR=0·1, gamma=2.5), NP (S=4, FDR=0·2, gamma=2.5), N2 (S=4, FDR=0·2, gamma=3), MP (S=5, FDR=0·15, gamma=2.5), and NS (S=5, FDR=0·15, gamma=2.5).

Supplementary results

Supplementary Table S1: Intra-subtype reassortment among Uganda H1N1pdm09 viruses

Event Count	Event ID	GiRaF Confidence	Run Freq (%)	Reassortment Candidate Viruses	Number of candidates	Sampling Date	Sampling Site	Segment pairs	Exchanged Segments	
									**Virus 1**	**Virus 2**
**1.**	410	1	100	ARU1544, ARU1548, EBB5261, EBB5309, EBB5389, KSW4567, KSW4584, KSW4597, KSW4719, MBA0906, NSY0055, NSY0059, NSY0064, TOR1642, TOR1744	15	June - November 2015, Feb 2016	Arua, Entebbe, Kawaala, Mbarara, Nsambya, Tororo	6	H1	PB2
**2.**	418	1	100						H1	PA
**3.**	496	1	100						H1	NP
**4.**	98	1	100						H1	N1
**5.**	205	1	100						H1	MP
**6.**	530	1	100						H1	NS
**7.**	478	0.99	100	ARU1080, FTL0867, FTL1119, FTL1128, FTL1130, KBK1430, MBA0689	7	August -November 2013, May - December 2014	Arua, Fort Portal, Koboko, Mbarara	7	H1	PA
**8.**	669	1	100						PB2	PB1
**9.**	337	1	100						PB2	PA
**10.**	386	1	100						PB2	NP
**11.**	243	1	100						PB2	N1
**12.**	389	1	100						PB2	MP
**13.**	176	1	100						PB2	NS
**14.**	159	1	100	ARU1544, ARU1548, EBB5261, EBB5309, EBB5389, KSW4567, KSW4584, KSW4597, KSW4719, MBA0906, NSY0055, NSY0059, NSY0064, TOR1642	14	June - November 2015	Arua, Entebbe, Kawaala, Mbarara, Nsambya, Tororo	6	PB2	PB1
**15.**	277	1	100						PB1	PA
**16.**	302	1	100						PB1	NP
**17.**	96	1	100						PB1	N1
**18.**	206	1	100						PB1	MP
**19.**	308	1	100						PB1	NS

Supplementary Table S1: Reassortment events and reassortants among whole genomes of 100 Uganda H1N1pdm09 viruses sampled between 2010 and 2018. Events and reassortants were detected by GiRaF(Nagarajan and Kingsford, 2011)

Supplementary Table S2: Intra-subtype reassortment among Uganda H3N2 viruses

Event Count	Event ID	GiRaF Confidence	Run Freq (x/20)	Run Freq (%)	Reassortant virus or set	Sampling date	Sampling site	Number of candidates	Involved Segments		Architecture	Unique Gene Pairs
									Virus 1	Virus 2		
**1.**	438	1	20	100	FTL1393	08 December 2015	Fort Portal	1	H3	PB2	PB1 H3 [PB2 PA NP N2 MP NS]	11
**2.**	388	1	20	100					H3	PA		
**3.**	452	1	20	100					H3	NP		
**4.**	370	1	20	100					H3	N2		
**5.**	464	1	20	100					H3	NS		
**6.**	245	1	20	100					PB2	PB1		
**7.**	341	1	20	100					PB1	PA		
**8.**	498	1	20	100					PB1	NP		
**9.**	387	1	20	100					PB1	N2		
**10.**	335	0·99	20	100					PB1	MP		
**11.**	406	1	20	100					PB1	NS		
**12.**	70	1	20	100	MBA1094	14 April 2017	Mbarara	1	H3	NP	NP N2 [PB2 PB1 PA H3 MP NS]	12
**13.**	42	1	20	100					H3	N2		
**14.**	241	1	20	100					PB2	NP		
**15.**	204	1	20	100					PB2	N2		
**16.**	51	1	20	100					PB1	NP		
**17.**	6	1	20	100					PB1	N2		
**18.**	94	1	20	100					PA	NP		
**19.**	68	1	20	100					PA	N2		
**20.**	71	1	20	100					NP	MP		
**21.**	150	1	20	100					NP	NS		
**22.**	33	0·99	20	100					N2	MP		
**23.**	365	1	20	100					N2	NS		
**24.**	252	0·99	20	100	EBB2779	29 September 2011	Entebbe	1	H3	NS	NS [PB2 PB1 PA H3 N2]	5
**25.**	220	0·99	20	100					PB2	NS		
**26.**	1912	0·99	20	100					PB1	NS		
**27.**	1362	0·99	20	100					PA	NS		
**28.**	316	0·99	20	100					N2	NS		
**29.**	519	1	20	100	EBB6461	15 June 2017	Entebbe	1	PB2	PB1	PB1 [PB2 NP MP]	3
**30.**	733	1	20	100					PB1	NP		
**31.**	477	1	20	100					PB1	MP		

Supplementary Table S2: Reassortment events and reassortants detected among whole genomes of 93 Uganda H3N2 viruses sampled between 2010 and 2018. Events and reassortants were detected by GiRaF(Nagarajan and Kingsford, 2011)**.**

Supplementary Table S3: Details on the distribution of H1N1pdm09 reassortant viruses with a specific architecture in Uganda and Africa between 2009 and 2020

		African H1N1pdm09 dataset (D2)			Ugandan H1N1pdm09 dataset (D1)		
**Count**	**Architecture**	**Country**	**Absolute number of reassortants (n)**	**Year of circulation**	**Site**	**Absolute number of reassortants (n)**	**Year of circulation**
1.	**N1 NS [PB2 PB1 PA H1]**	Ethiopia	3	2009			
		Mali	1	2009			
		CoteD'iviore	1	2009			
		Egypt	1	2009			
		Kenya	7	2009			
		Seychelles	1	2009			
		Tanzania	1	2009			
		Uganda	1	2009			
		Ethiopia	1	2010			
		Kenya	2	2010			
		Uganda	13	2010			
		Uganda	4	2011			
2.	**N1 NS [PB2 PB1 PA H1 MP]**	Kenya	1	2010			
3.	**PB2 PB1 PA H1 MP NS [N1]**	Kenya	1	2010			
4.	**PB2 PB1 PA H1 NS [N1]**	Kenya	1	2010			
5.	**PB2 PB1 PA NS [H1 N1]**	Kenya	5	2010			
		Uganda	6	2011			
6.	**NS [PB2 PB1 H1]**	Kenya	1	2011			
		Uganda	8	2011			
		Uganda	6	2013			
7.	**PB2 [PB1 PA H1 NP N1 MP NS]**				Arua	1	2013
					Koboko	1	2013
					Fort Portal	4	2014
					Mbarara	1	2014
8.	**PB2 NS [PB1 PA H1 NP N1 MP]**	Uganda	2	2013			
		Uganda	7	2014			
9.	**NS [PB2 PB1 PA H1 NP N1]**	Uganda	3	2014			
10.	**PB2 PB1 PA H1 NP N1 MP NS []**	South Africa	1	2015			
11.	**PB1 H1 [PB2 PA NP N1 MP NS]**	Uganda	14	2015	Arua	2	2015
		Uganda	2	2016	Entebbe	3	2015
					Kawaala	4	2015
					Mbarara	1	2015
					Nsambya	3	2015
					Tororo	1	2015
12.	**H1 [PB2 PB1 PA NP N1 MP NS]**				Tororo	1	2016
13.	**H1 [PB2 PA N1]**	Mozambique	3	2016			
14.	**MP NS [PB2 PB1 PA H1 NP N1]**	Madagascar	1	2016			
15.	**NP [PB2 PB1 PA H1 N1 MP NS]**	Mali	1	2016			
16.	**NP [PB2 PB1 H1 N1 NS]**	Kenya	1	2018			
17.	**PA [PB1 H1 NP N1 MP]**	Nigeria	1	2018			

Supplementary Table S3: Temporal and spatial distribution of H1N1pdm09 reassortant viruses with a specific architecture in Uganda and Africa between 2009 and 2020.

Supplementary Table S4: Details on the distribution of H3N2 reassortant viruses with a specific architecture in Uganda and Africa between 1994 and 2020

		African H3N2 dataset (D2)			Ugandan H3N2 dataset (D1)		
**Count**	**Architecture**	**Country**	**Absolute number of reassortants (n)**	**Year of circulation**	**Site**	**Absolute number of reassortants (n)**	**Year of circulation**
1.	**MP [PB2 PB1 PA H3 NP N2 NS]**	Kenya	1	2011			
2.	**PB2 PA MP [PB1 H3 NP N2 NS]**	Kenya	1	2011			
3.	**NS [PB2 PB1 PA H3 N2]**				Entebbe	1	2011
4.	**H3 [PB1 PA N2 MP NS]**	Uganda	1	2013			
		Kenya	3	2014			
		Uganda	1	2015			
		Uganda	1	2016			
5.	**MP [PB2 PB1 PA H3 NP N2]**	Tanzania	1	2014			
6.	**PB2 H3 NP [PB1 N2 MP]**	Tanzania	21	2014			
7.	**PB2 PB1 PA H3 NP N2 MP [NS]**	Tanzania	1	2014			
8.	**PB2 NP [PB1 H3 N2 MP]**	Ethiopia	9	2014			
		Nigeria	1	2014			
		Tanzania	1	2014			
		Uganda	1	2014			
		Madagascar	2	2015			
		Uganda	1	2015			
9.	**PB1 H3 [PB2 PA NP N2 NS]**	Uganda	1	2015			
10.	**PB1 H3 [PB2 PA NP N2 MP NS]**				Fort Portal	1	2015
11.	**H3 [PB2 PB1 PA NP N2]**	**Mali**	1	2015			
12.	**MP NS [PB2 PA H3 NP]**	Kenya	1	2015			
13.	**NP [PB2 PA H3 N2]**	Burkina Faso	1	2015			
14.	**NS [PB2 PB1 PA H3 NP N2]**	Kenya	1	2015			
		Mali	1	2015			
		Kenya	1	2016			
15.	**H3 NP [PB2 PB1 PA N2 NS]**	Nigeria	1	2015			
		Kenya	1	2016			
		South Africa	1	2016			
		Uganda	2	2016			
		Madagascar	13	2017			
		CoteD'iviore	4	2017			
		Ethiopia	9	2017			
		Kenya	5	2017			
		Niger	10	2017			
		Sierra Leone	2	2017			
		South Africa	3	2017			
		Tanzania	28	2017			
		Togo	8	2017			
		Burkina Faso	4	2018			
		Congo	17	2018			
		Kenya	3	2018			
		Mali	4	2018			
		Nigeria	8	2018			
		Tanzania	1	2018			
		Burkina Faso	4	2019			
		CoteD'iviore	2	2019			
		Mali	21	2019			
		Niger	8	2019			
		Nigeria	3	2019			
		Togo	4	2019			
16.	**PB2 [PB1 PA H3 NP N2 MP NS]**	Kenya	1	2016			
17.	**H3 PA [PB2 PB1 NP N2 MP NS]**	Kenya	1	2016			
18.	**H3 [PB2 PB1 PA NP N2 MP]**	Uganda	3	2016			
		Kenya	1	2017			
		South Africa	1	2017			
		Uganda	5	2017			
19.	**NP N2 [PB2 PB1 PA H3 MP NS]**	Uganda	2	2017	Mbarara	1	2017

20.	**PB1 [PB2 NP MP]**					Entebbe	1	2017
21.	**NS [PB2 PA NP]**	Uganda	1	2017				
22.	**PA [PB2 PB1 H3 NP N2 NS]**	Kenya	1	2017				
23.	**PB1 [PB2 PA H3 NP N2 MP]**	Uganda	1	2017				
24.	**H3 MP [PB2 PB1 PA NP]**	Kenya	14	2018				
		Kenya	13	2019				
25.	**PA H3 NP N2 NS [PB2 PB1 MP]**	Burkina Faso	3	2018				
		Congo	12	2018				
		Congo	11	2019				
26.	**N2 NS [PB2 PB1 PA H3 NP MP]**	Togo	1	2019				
27.	**NS [PB2 PB1 PA H3 NP N2 MP]**	Mozambique	1	2019				
28.	**PA H3 N2 [PB2 PB1 NP MP NS]**	Mozambique	7	2019				
29.	**PB1 H3 MP [PB2 PA NP N2 NS]**	Togo	2	2019				
30.	**PB2 PB1 H3 NP N2 MP NS [PA]**	CoteD'iviore	4	2019				
31.	**PA [PB2 PB1 NP N2]**	Burkina Faso	13	2019				
		CoteD'iviore	17	2019				
		Mali	26	2019				
		Niger	31	2019				
		Nigeria	28	2019				
		Togo	51	2019				
		Congo	1	2020				
		Nigeria	1	2020				

Supplementary Table S4: Temporal and spatial distribution of H3N2 reassortant viruses with a specific architecture in Uganda and Africa between 1994 and 2020

Supplementary Table S5: Intra-subtype reassortment events and reassortants among Africa H1N1pdm09 viruses

Event Count	Event ID	Segment pair	GiRaF Confidence	Candidate reassortant set	Number of reassortants
1.	**7865**	PB2, PB1	0·99	ARU0581 ARU0582 EBB2780 EPIISL140406 EPIISL140407 EPIISL140408 EPIISL140409 EPIISL140410 EPIISL140411 EPIISL140412 KBK0655 KSW2793 KSY0539	13
2.	**10470**	PB2, PA	0·99		
3.	**11775**	PB1, PA	0·99		
4.	**9235**	PA, N1	0·99		
5.	**8753**	PB2, PB1	1	ARU1080 EPIISL176803 EPIISL176806 FTL0867 FTL1119 FTL1128 FTL1130 KBK1430 MBA0689	9
6.	**9722**	PB2, PA	1		
7.	**11465**	PB2, NP	1		
8.	**8861**	PB2, N1	1		
9.	**7154**	PB2, MP	1		
10.	**153**	PB2, NS	1		
11.	**12260**	H1, PA	1		
12.	**7617**	PB2, PB1	1	ARU1544 ARU1548 EBB5261 EBB5309 EBB5389 KSW4567 KSW4584 KSW4597 KSW4719 MBA0906 NSY0055 NSY0059 NSY0064 TOR1642	14
13.	**8562**	PB1, PA	1		
14.	**10326**	PB1, NP	1		
15.	**8545**	PB1, N1	1		
16.	**6664**	PB1, NS	1		
17.	**7587**	PB2, N1	0·99	ARU0578 ARU0587 EBB2451 EBB2480 EBB2484 EBB2494 EBB2497 EBB2498 EBB2516 EBB2521 EBB2707 EPIISL139708 EPIISL139716 EPIISL139719 EPIISL139720 EPIISL140395 EPIISL140396 EPIISL140404 EPIISL140405 EPIISL140413 EPIISL33576 EPIISL34539 EPIISL35013 EPIISL35021 EPIISL35022 EPIISL62231 EPIISL62338 EPIISL71367 EPIISL78853 EPIISL78854 EPIISL78855 KBK0372 KBK0377 KSY0444 KSY0445 KSY0496 KSY0533	37
18.	**8609**	PB1, N1	1		
19.	**7224**	PA, N1	1		
20.	**6198**	PB2, MP	1	EPIISL140406	1
21.	**5403**	PB1, MP	1		
22.	**7317**	PA, MP	0.99		
23.	**8224**	PB2, NS	0·99	ARU0578 ARU0581 ARU0582 ARU0587 ARU1080 EBB2451 EBB2480 EBB2484 EBB2494 EBB2497 EBB2498 EBB2516 EBB2521 EBB2653 EBB2669 EBB2693 EBB2694 EBB2707 EBB2780 EBB2904 EBB3824 EPIISL106806 EPIISL139708 EPIISL139716 EPIISL139719 EPIISL139720 EPIISL140395 EPIISL140396 EPIISL140404 EPIISL140405 EPIISL140406 EPIISL140407 EPIISL140408 EPIISL140409 EPIISL140410 EPIISL140411 EPIISL140412 EPIISL140413 EPIISL176803 EPIISL176806 EPIISL33576 EPIISL34539 EPIISL35013 EPIISL35021 EPIISL35022 EPIISL62231 EPIISL62338 EPIISL71367 EPIISL78853 EPIISL78854 EPIISL78855 FTL0151 FTL0867 FTL1119 FTL1128 FTL1130 KBK0372 KBK0377 KBK0655 KBK1430 KIS0338 KSW2788 KSW2793 KSW3485 KSW3539 KSW3548 KSW3711 KSY0444 KSY0445 KSY0496 KSY0533 KSY0539 KSY0843 MBA0689	74
24.	**7659**	PB1, NS	1		
25.	**7107**	H1, NS	1		
26.	**149**	PB2, NS	0.99	EBB4743 KIS1560 KIS1715	3
27.	**248**	PB1, NS	1		
28.	**44**	PA, NS	1		
29.	**48**	H1, NS	0.99		
30.	**153**	NP, NS	0.99		
31.	**11**	N1, NS	0.99		
32.	**6160**	H1, NS	1	EPIISL140413	1
33.	**7365**	PB2, NS	0.99		
34.	**4465**	MP, NS	0.99		
35.	**10344**	PB1, NP	0·94	ARU1544 ARU1548 EBB5261 EBB5309 EBB5389 EPIISL227473 KSW4567 KSW4584 KSW4597 KSW4719 MBA0906 NSY0055 NSY0059 NSY0064 TOR1642 TOR1744	16
36.	**10070**	H1, PB2	1		
37.	**11458**	H1, PA	1		
38.	**8698**	H1, NP	1		
39.	**7503**	H1, N1	1		
40.	**5897**	H1, MP	1		
41.	**7567**	H1, NS	1		
42.	**5840**	H1, PB2	1	EPIISL207384	1
43.	**6940**	H1, PB1	1		
44.	**6672**	PB2, PA	1		
45.	**6995**	PB2, NP	1		
46.	**6185**	PB2, N1	1		
47.	**3340**	PB2, NS	1		
48.	**7295**	PB1, PA	1		
49.	**8586**	PB1, NP	1		
50.	**7430**	PB1, N1	1		
51.	**3334**	PB1, NS	1		
52.	**2286**	MP, NS	1		
53.	**9328**	H1, PB2	1	EPIISL140406 EPIISL140408	2
54.	**9598**	H1, PB1	1		
55.	**10605**	H1, PA	1		
56.	**4967**	H1, PB2	0.99	EPIISL236216 EPIISL236217 EPIISL236218	3
57.	**6965**	H1, PA	1		
58.	**4350**	H1, N1	0.99		
59.	**10284**	H1, PB1	1	EPIISL227473 TOR1744	2
60.	**5711**	H1, NS	0.98		
61.	**5**	PB1, PA	0.99		
62.	**847**	H1, PA	1	EPIISL353346	1
63.	**1763**	PB1, PA	1		
64.	**1092**	PA, NP	1		
65.	**1422**	PA, N1	1		
66.	**1507**	PA, MP	0.97		
67.	**6515**	H1, NP	1	EPIISL232676	1
68.	**5136**	PB2, NP	1		
69.	**6355**	PB1, NP	1		
70.	**6776**	PA, NP	1		
71.	**5457**	NP, N1	1		
72.	**4158**	NP, MP	1		
73.	**3766**	NP, NS	1		
74.	**960**	H1, NP	0.98	EPIISL335908	1
75.	**2725**	PB2, NP	0.99		
76.	**1340**	PB1, NP	0.99		
77.	**2659**	NP, N1	0.99		
78.	**2000**	NP, NS	0.98		
79.	**5524**	H1, MP	1	EPIISL207384 EPIISL237813	2

80.	**6392**	PA, MP	1		
81.	**7306**	NP, MP	1		
82.	**4939**	N1, MP	1		
83.	**3533**	H1, MP	1	EPIISL237813	1
84.	**3733**	H1, NS	1		
85.	**3579**	PB2, MP	1		
86.	**4057**	PB2, NS	1		
87.	**3573**	PB1, MP	1		
88.	**4300**	PB1, NS	1		
89.	**4253**	PA, MP	1		
90.	**4766**	PA, NS	1		
91.	**4437**	NP, MP	1		
92.	**3997**	NP, NS	1		
93.	**2984**	N1, MP	1		
94.	**3233**	N1, NS	1		

Supplementary Table S5: Reassortment events and reassortants detected among whole genomes of 758 Africa H1N1pdm09 viruses sampled between 2009 and 2020. Events and reassortants were detected by GiRaF (Nagarajan and Kingsford, 2011).

Supplementary Table S6: Intra-subtype reassortment events and reassortants among Africa H3N2 viruses

Event Count	Event ID	Segment pair	GiRaF Confidence	Reassortment candidate viruses	Number of reassortants in set
1.	**12170**	PB2, NP	0.99	ARU1409 EPIISL171352 EPIISL171378 EPIISL171380 EPIISL172531 EPIISL172532 EPIISL172592 EPIISL172733 EPIISL172737 EPIISL172750 EPIISL172752 EPIISL172755 EPIISL172759 EPIISL172779 EPIISL172781 EPIISL172785 EPIISL172787 EPIISL172789 EPIISL172796 EPIISL172797 EPIISL172800 EPIISL172803 EPIISL172808 EPIISL172811 EPIISL172814 EPIISL174120 EPIISL174126 EPIISL174127 EPIISL174137 EPIISL175202 EPIISL175205 EPIISL188871 EPIISL192158 EPIISL206244 EPIISL207448 FTL1327	36
2.	**11239**	PB2, MP	0.99		
3.	**14591**	PB1, NP	1		
4.	**14303**	NP, N2	1		
5.	**21367**	H3, PB2	0.99		
6.	**21152**	H3, PB1	0.99		
7.	**26975**	H3, PB2	0.98	EBB6093 EPIISL255342 EPIISL286128 EPIISL335644 EPIISL336501 EPIISL336502 EPIISL336503 EPIISL336504 EPIISL338561 EPIISL339852 EPIISL341025 EPIISL346059 EPIISL346060 EPIISL346061 EPIISL346062 EPIISL362214 EPIISL362217 EPIISL362221 EPIISL362222 EPIISL362225 EPIISL362226 EPIISL362227 EPIISL362228 EPIISL362231 EPIISL363896 EPIISL363898 EPIISL364540 EPIISL364541 EPIISL377651 EPIISL381598 EPIISL394949 EPIISL394950 EPIISL417899 KIS2305 KIS2541 KSW5444 KSW5495 KSW5544	38
8.	**28334**	H3, PB1	0.98		
9.	**21722**	H3, PA	0.98		
10.	**23063**	H3, NP	0.99		
11.	**16954**	PB2, PB1	1	EBB6093 EPIISL255342 EPIISL286128 EPIISL381598 KIS2305 KIS2541 KSW5444 KSW5495 KSW5544	9
12.	**240**	H3, PB2	1		
13.	**19733**	H3, PB1	1		
14.	**21575**	H3, PA	0.99		
15.	**19202**	H3, NP	0.99		
16.	**36736**	H3, N2	1		
17.	**9373**	H3, MP	1		
18.	**3709**	PB2, PB1	1	EBB6461	1
19.	**4328**	PB1, PA	1		
20.	**3545**	PB1, NP	1		
21.	**4144**	PB1, N2	1		
22.	**2941**	PB1, MP	1		
23.	**3310**	H3, PB1	1		
24.	**8706**	MP, NS	0.99	EPIISL171375 EPIISL171377 EPIISL393708 NSY0304 TOR0492 TOR1664	6
25.	**1414**	H3, PB1	1		
26.	**1850**	H3, PA	0.99		
27.	**3325**	H3, N2	0.99		
28.	**2047**	H3, MP	0.99		
29.	**26866**	H3, PB2	0.97	EPIISL172737 EPIISL172750 EPIISL172752 EPIISL172755 EPIISL172759 EPIISL172781 EPIISL172785 EPIISL172787 EPIISL172789 EPIISL172796 EPIISL172797 EPIISL172800 EPIISL172803 EPIISL172811 EPIISL172814 EPIISL174120 EPIISL174126 EPIISL174127 EPIISL175202 EPIISL175205 EPIISL188871	21
30.	**28211**	H3, PB1	0.99		
31.	**37166**	H3, N2	0.99		
32.	**12858**	H3, MP	0.99		
33.	**10673**	PB1, MP	1	EPIISL172774	1
34.	**8719**	N2, MP	1		
35.	**10067**	H3, MP	0.99		
36.	**9735**	PB2, MP	1	EPIISL172774 EPIISL174124	2
37.	**11690**	PA, MP	1		
38.	**8067**	NP, MP	1		
39.	**10512**	H3, MP	1		
40.	**11223**	PB2, MP	0.99	EPIISL172774 EPIISL174124 EPIISL335644 EPIISL336501 EPIISL336502 EPIISL336503 EPIISL336504 EPIISL338561 EPIISL339852 EPIISL341025 EPIISL346059 EPIISL346060 EPIISL346061 EPIISL346062 EPIISL362214 EPIISL362217 EPIISL362221 EPIISL362222 EPIISL362225 EPIISL362226 EPIISL362227 EPIISL362228 EPIISL362231 EPIISL363896 EPIISL363898 EPIISL364540 EPIISL364541 EPIISL377651 EPIISL394949 EPIISL394950 EPIISL417899	31
41.	**11696**	PA, MP	1		
42.	**8073**	NP, MP	0.99		
43.	**17745**	PB2, PB1	1	EPIISL174124	1
44.	**19688**	PB2, N2	1		
45.	**21628**	PB1, PA	1		
46.	**15921**	PB1, NP	1		
47.	**22234**	PA, N2	1		
48.	**16872**	NP, NS	0.99		
49.	**14243**	NP, N2	1		
50.	**21116**	H3, PB1	1		
51.	**18509**	H3, NS	0.99		
52.	**28680**	H3, N2	1		
53.	**14656**	PB2, NS	1	EPIISL206195	1
54.	**17256**	PB1, NS	1		
55.	**16749**	PA, NS	1		
56.	**13627**	NP, NS	0.99		
57.	**15901**	N2, NS	1		
58.	**16678**	H3, NS	1		
59.	**17513**	H3, PB2	1	EPIISL207422	1
60.	**18594**	H3, PB1	1		
61.	**14921**	H3, PA	1		
62.	**17830**	H3, NP	0.99		
63.	**25999**	H3, N2	0.99		
64.	**12095**	PB2, NP	1	EPIISL211919	1
65.	**14522**	PB1, NP	1		
66.	**14701**	PA, NP	1		
67.	**12029**	NP, NS	1		
68.	**10544**	NP, N2	1		
69.	**14403**	H3, PB2	1		
70.	**12484**	H3, PA	1		
71.	**13894**	H3, NS	1		
72.	**21396**	H3, N2	1		
73.	**11720**	PB2, NP	0.99	EPIISL220283 EPIISL232562 EPIISL268306 EPIISL268307 EPIISL268308 EPIISL268309 EPIISL268310 EPIISL268311 EPIISL268312 EPIISL268313 EPIISL268314 EPIISL268315 EPIISL268316 EPIISL268317 EPIISL268318 EPIISL268319 EPIISL268320 EPIISL268321 EPIISL268322 EPIISL268323 EPIISL268324 EPIISL268325 EPIISL268326 EPIISL268327 EPIISL268331 EPIISL268332 EPIISL270211 EPIISL270212 EPIISL270213 EPIISL273620 EPIISL273663 EPIISL273665 EPIISL273667 EPIISL273669 EPIISL273671 EPIISL273686 EPIISL275932 EPIISL277470 EPIISL281757 EPIISL281817 EPIISL282861 EPIISL283312 EPIISL288511 EPIISL288512 EPIISL288513 EPIISL288515 EPIISL288516 EPIISL288517 EPIISL288518 EPIISL288519 EPIISL290658 EPIISL290659 EPIISL290660 EPIISL290661 EPIISL292535 EPIISL292537 EPIISL292538 EPIISL292539 EPIISL296085 EPIISL298381 EPIISL299831 EPIISL299832 EPIISL299833 EPIISL299834 EPIISL299836 EPIISL299840 EPIISL299842 EPIISL299843 EPIISL299852 EPIISL299853 EPIISL299893 EPIISL299894 EPIISL300773 EPIISL300779 EPIISL300840 EPIISL303250 EPIISL305003 EPIISL306196 EPIISL306260 EPIISL319709 EPIISL320281 EPIISL320310 EPIISL320344 EPIISL320345 EPIISL320346 EPIISL320347 EPIISL320348 EPIISL320349 EPIISL320351 EPIISL321915 EPIISL322946 EPIISL329853 EPIISL329854 EPIISL330475 EPIISL331507 EPIISL335648 EPIISL335656 EPIISL346055 EPIISL346064 EPIISL347944 EPIISL348142 EPIISL348146 EPIISL351879 EPIISL351989 EPIISL352057 EPIISL353449 EPIISL353514 EPIISL353702 EPIISL355536 EPIISL355540 EPIISL355604 EPIISL355764 EPIISL356223 EPIISL356224 EPIISL356225 EPIISL356294 EPIISL356301 EPIISL356312 EPIISL357855 EPIISL362170 EPIISL362177 EPIISL362389 EPIISL362391 EPIISL362394 EPIISL362553 EPIISL363855 EPIISL363866 EPIISL363867 EPIISL363869 EPIISL363870 EPIISL363874 EPIISL363881 EPIISL363888 EPIISL363892 EPIISL363907 EPIISL363934 EPIISL363937 EPIISL363978 EPIISL364005 EPIISL364010 EPIISL365782 EPIISL365783 EPIISL377868 EPIISL377872 EPIISL380152 EPIISL392520 EPIISL392524 EPIISL392529 EPIISL393572 EPIISL393574 EPIISL393575 EPIISL393576 EPIISL393577 EPIISL393579 EPIISL393581 EPIISL393582 EPIISL393604 EPIISL393718 EPIISL393723 EPIISL393747 EPIISL393751 EPIISL393953 EPIISL393967 EPIISL397182 EPIISL398314 EPIISL398532 EPIISL491766 KSW5031 TOR1716	169
74.	**13296**	PA, NP	0.99		
75.	**12509**	NP, NS	0.97		
76.	**24023**	H3, N2	0.99		
77.	**1553**	H3, PA	1	EPIISL232562 EPIISL268306 EPIISL268307 EPIISL268308 EPIISL268309 EPIISL268310 EPIISL268311 EPIISL268312 EPIISL268313 EPIISL268314 EPIISL268315 EPIISL268316 EPIISL268317 EPIISL268318 EPIISL268319 EPIISL268320 EPIISL268321 EPIISL268322 EPIISL268323 EPIISL268324 EPIISL268325 EPIISL268326 EPIISL268327 EPIISL268331 EPIISL268332 EPIISL270211 EPIISL270212 EPIISL270213 EPIISL273620 EPIISL273663 EPIISL273665 EPIISL273667 EPIISL273669 EPIISL273671 EPIISL273686 EPIISL275932 EPIISL277470 EPIISL281757 EPIISL281817 EPIISL282861 EPIISL283312 EPIISL288511 EPIISL288512 EPIISL288513 EPIISL288515 EPIISL288516 EPIISL288517 EPIISL288518 EPIISL288519 EPIISL290658 EPIISL290659 EPIISL290660 EPIISL290661 EPIISL292535 EPIISL292537 EPIISL292538 EPIISL292539 EPIISL296085 EPIISL298381 EPIISL299831 EPIISL299832 EPIISL299833 EPIISL299834 EPIISL299836 EPIISL299840 EPIISL299842 EPIISL299843 EPIISL299852 EPIISL299853 EPIISL299893 EPIISL299894 EPIISL300773 EPIISL300779 EPIISL300840 EPIISL303250 EPIISL305003 EPIISL306196 EPIISL306260 EPIISL319709 EPIISL320281 EPIISL320310 EPIISL320344 EPIISL320345 EPIISL320346 EPIISL320347 EPIISL320348 EPIISL320349 EPIISL320351 EPIISL321915 EPIISL322946 EPIISL329853 EPIISL329854 EPIISL330475 EPIISL331507 EPIISL335648 EPIISL335656 EPIISL346055 EPIISL346056 EPIISL346057 EPIISL346064 EPIISL346220 EPIISL347943 EPIISL347944 EPIISL347947 EPIISL347948 EPIISL347950 EPIISL347951 EPIISL347952 EPIISL348142 EPIISL348146 EPIISL348156 EPIISL348160 EPIISL349707 EPIISL349708 EPIISL349709 EPIISL351879 EPIISL351920 EPIISL351924 EPIISL351989 EPIISL352057 EPIISL353449 EPIISL353514 EPIISL353702 EPIISL355536 EPIISL355540 EPIISL355604 EPIISL355764 EPIISL356223 EPIISL356224 EPIISL356225 EPIISL356294 EPIISL356301 EPIISL356312 EPIISL357855 EPIISL362170 EPIISL362177 EPIISL362389 EPIISL362391 EPIISL362394 EPIISL362395 EPIISL362396 EPIISL362553 EPIISL363855 EPIISL363866 EPIISL363867 EPIISL363869 EPIISL363870 EPIISL363874 EPIISL363880 EPIISL363881 EPIISL363884 EPIISL363888 EPIISL363892 EPIISL363907 EPIISL363934 EPIISL363937 EPIISL363978 EPIISL364005 EPIISL364010 EPIISL365782 EPIISL365783 EPIISL377686 EPIISL377868 EPIISL377872 EPIISL377873 EPIISL380152 EPIISL380153 EPIISL392520 EPIISL392524 EPIISL392529 EPIISL393572 EPIISL393574 EPIISL393575 EPIISL393576 EPIISL393577 EPIISL393579 EPIISL393581 EPIISL393582 EPIISL393604 EPIISL393718 EPIISL393723 EPIISL393747 EPIISL393751 EPIISL393752 EPIISL393953 EPIISL393967 EPIISL397182 EPIISL398314 EPIISL398532 EPIISL491766 KSW5031 TOR1716	192
78.	**13921**	H3, NS	1		
79.	**21487**	H3, N2	1		

80.	**17401**	PB1, N2	1	EPIISL232562 EPIISL268306 EPIISL268307 EPIISL268308 EPIISL268309 EPIISL268310 EPIISL268311 EPIISL268312 EPIISL268313 EPIISL268314 EPIISL268315 EPIISL268316 EPIISL268317 EPIISL268318 EPIISL268319 EPIISL268320 EPIISL268321 EPIISL268322 EPIISL268323 EPIISL268324 EPIISL268325 EPIISL268326 EPIISL268327 EPIISL268331 EPIISL268332 EPIISL270211 EPIISL270212 EPIISL270213 EPIISL273620 EPIISL273663 EPIISL273665 EPIISL273667 EPIISL273669 EPIISL273671 EPIISL273686 EPIISL275932 EPIISL277470 EPIISL281757 EPIISL281817 EPIISL282861 EPIISL283312 EPIISL288511 EPIISL288512 EPIISL288513 EPIISL288515 EPIISL288516 EPIISL288517 EPIISL288518 EPIISL288519 EPIISL290658 EPIISL290659 EPIISL290660 EPIISL290661 EPIISL292535 EPIISL292537 EPIISL292538 EPIISL292539 EPIISL296085 EPIISL298381 EPIISL299831 EPIISL299832 EPIISL299833 EPIISL299834 EPIISL299836 EPIISL299840 EPIISL299842 EPIISL299843 EPIISL299852 EPIISL299853 EPIISL299893 EPIISL299894 EPIISL300773 EPIISL300779 EPIISL300840 EPIISL303250 EPIISL305003 EPIISL306196 EPIISL306260 EPIISL319709 EPIISL320281 EPIISL320310 EPIISL320344 EPIISL320345 EPIISL320346 EPIISL320347 EPIISL320348 EPIISL320349 EPIISL320351 EPIISL321915 EPIISL322946 EPIISL329853 EPIISL329854 EPIISL330475 EPIISL331507 EPIISL335648 EPIISL335656 EPIISL346055 EPIISL346064 EPIISL347944 EPIISL348142 EPIISL348146 EPIISL351879 EPIISL351989 EPIISL352057 EPIISL353449 EPIISL353514 EPIISL353702 EPIISL355536 EPIISL355540 EPIISL355604 EPIISL355764 EPIISL356223 EPIISL356224 EPIISL356225 EPIISL356294 EPIISL356301 EPIISL356312 EPIISL357855 EPIISL362170 EPIISL362177 EPIISL362389 EPIISL362391 EPIISL362394 EPIISL362553 EPIISL363855 EPIISL363866 EPIISL363867 EPIISL363869 EPIISL363870 EPIISL363874 EPIISL363881 EPIISL363888 EPIISL363892 EPIISL363907 EPIISL363934 EPIISL363937 EPIISL363978 EPIISL364005 EPIISL364010 EPIISL365782 EPIISL365783 EPIISL377868 EPIISL377872 EPIISL380152 EPIISL392520 EPIISL392524 EPIISL392529 EPIISL393572 EPIISL393574 EPIISL393575 EPIISL393576 EPIISL393577 EPIISL393579 EPIISL393581 EPIISL393582 EPIISL393604 EPIISL393718 EPIISL393723 EPIISL393747 EPIISL393751 EPIISL393953 EPIISL393967 EPIISL397182 EPIISL398314 EPIISL398532 EPIISL491766 KSW5031 TOR1716	168
81.	**11970**	PA, NP	0.98		
82.	**20658**	H3, N2	1		
83.	**18427**	PB1, NP	0.99	EPIISL255341 EPIISL346056 EPIISL346057 EPIISL346220 EPIISL347943 EPIISL347947 EPIISL347948 EPIISL347950 EPIISL347951 EPIISL347952 EPIISL348156 EPIISL348160 EPIISL349707 EPIISL349708 EPIISL349709 EPIISL351920 EPIISL351924 EPIISL362395 EPIISL362396 EPIISL363880 EPIISL363884 EPIISL377686 EPIISL377873 EPIISL380153 MBA1094	25
84.	**27214**	PB1, N2	0.99		
85.	**21126**	PA, N2	0.99		
86.	**10700**	PB2, NP	1	EPIISL255341 MBA1094	2
87.	**14945**	PB2, N2	1		
88.	**11374**	PB1, NP	1		
89.	**15239**	PB1, N2	1		
90.	**11734**	PA, NP	1		
91.	**14638**	PA, N2	1		
92.	**6256**	NP, MP	0.99		
93.	**3693**	N2, NS	1		
94.	**6796**	N2, MP	1		
95.	**14176**	H3, NP	1		
96.	**19339**	H3, N2	1		
97.	**790**	PB2, NS	1	EPIISL281755	1
98.	**2115**	PA, NS	1		
99.	**2703**	NP, NS	1		
100.	**4970**	NP, MP	0.99	EPIISL281802	1
101.	**20825**	H3, PB2	0.99		
102.	**10641**	H3, PA	1		
103.	**6064**	H3, NP	1		
104.	**17823**	H3, N2	1		
105.	**25179**	H3, PB1	1	EPIISL281802 EPIISL363896 EPIISL363898	3
106.	**19846**	H3, PA	0.99		
107.	**33860**	H3, N2	0.99		
108.	**7175**	PB2, NP	0.99	EPIISL320281 EPIISL320310 EPIISL320344 EPIISL320345 EPIISL320346 EPIISL320347 EPIISL320348 EPIISL320349 EPIISL320351 EPIISL321915 EPIISL322946 EPIISL329853 EPIISL329854 EPIISL346055 EPIISL347944 EPIISL351989 EPIISL380152	17
109.	**8286**	PA, NP	0.99		
110.	**9654**	H3, NP	0.99		
111.	**19226**	H3, PB2	0.99	EPIISL335644 EPIISL336501 EPIISL336502 EPIISL336503 EPIISL336504 EPIISL338561 EPIISL339852 EPIISL341025 EPIISL346059 EPIISL346060 EPIISL346061 EPIISL346062 EPIISL362214 EPIISL362217 EPIISL362221 EPIISL362222 EPIISL362225 EPIISL362226 EPIISL362227 EPIISL362228 EPIISL362231 EPIISL363896 EPIISL363898 EPIISL364540 EPIISL364541 EPIISL377651 EPIISL394949 EPIISL394950 EPIISL417899	29
112.	**19760**	H3, PB1	0.99		
113.	**16614**	H3, PA	0.99		
114.	**18631**	H3, NP	1		
115.	**6617**	PB2, NP	1	EPIISL346056 EPIISL346057 EPIISL346220 EPIISL347943 EPIISL347947 EPIISL347948 EPIISL347950 EPIISL347951 EPIISL347952 EPIISL348156 EPIISL348160 EPIISL349707 EPIISL349708 EPIISL349709 EPIISL351920 EPIISL351924 EPIISL362395 EPIISL362396 EPIISL363880 EPIISL363884 EPIISL377686 EPIISL377873 EPIISL380153	23
116.	**8498**	PB2, N2	1		
117.	**8002**	PB1, NP	1		
118.	**10106**	PB1, N2	1		
119.	**7321**	PA, NP	1		
120.	**8537**	PA, N2	1		
121.	**7885**	NP, NS	1		
122.	**4789**	NP, MP	0.99		
123.	**8959**	N2, NS	1		
124.	**4181**	N2, MP	1		
125.	**9488**	H3, NP	1		
126.	**13097**	H3, N2	1		
127.	**4486**	PB2, NS	1	EPIISL355604 EPIISL356301 EPIISL363937	3
128.	**5815**	PB1, NS	1		
129.	**5963**	PA, NS	1		
130.	**4532**	NP, NS	1		
131.	**6063**	N2, NS	1		
132.	**3720**	MP, NS	1		
133.	**4908**	H3, NS	1		
134.	**5426**	PB2, NS	1	EPIISL355604 EPIISL356301 EPIISL363937 EPIISL365784 EPIISL365785 EPIISL390030 EPIISL393570 EPIISL400779	8
135.	**6371**	NP, NS	1		
136.	**3820**	MP, NS	0.99		
137.	**7582**	PB1, NS	1	EPIISL355604 EPIISL356301 EPIISL363937 EPIISL400779	4
138.	**8187**	PA, NS	0.99		
139.	**6161**	H3, NS	0.99		
140.	**4808**	PB2, PA	0.99	EPIISL363873 EPIISL363876 EPIISL363894 EPIISL393757 EPIISL393758 EPIISL393759 EPIISL394904 EPIISL397199 EPIISL398311 EPIISL398313 EPIISL398535 EPIISL398555 EPIISL398790 EPIISL398795 EPIISL398945 EPIISL400781 EPIISL400782 EPIISL400789 EPIISL400790 EPIISL400791 EPIISL400792 EPIISL402407 EPIISL402502 EPIISL402504 EPIISL402506 EPIISL402508 EPIISL402765 EPIISL402766 EPIISL402767 EPIISL402768 EPIISL402769 EPIISL402771 EPIISL402772 EPIISL402773 EPIISL402774 EPIISL402775 EPIISL402776 EPIISL402779 EPIISL402780 EPIISL409091 EPIISL409343 EPIISL409344 EPIISL409346 EPIISL409347 EPIISL409348 EPIISL409349 EPIISL409350 EPIISL409351 EPIISL409353 EPIISL409354 EPIISL409355 EPIISL409356 EPIISL409357 EPIISL409358 EPIISL409359 EPIISL409360 EPIISL409361 EPIISL409363 EPIISL409371 EPIISL409407 EPIISL409410 EPIISL409411 EPIISL409412 EPIISL409413 EPIISL409414 EPIISL409415 EPIISL409416 EPIISL409417 EPIISL409437 EPIISL409449 EPIISL413231 EPIISL413240 EPIISL413266 EPIISL413713 EPIISL413715 EPIISL413717 EPIISL413719 EPIISL413720 EPIISL413721 EPIISL413722 EPIISL413723 EPIISL413724 EPIISL413725 EPIISL413726 EPIISL413727 EPIISL413733 EPIISL413734 EPIISL413737 EPIISL413741 EPIISL413742 EPIISL418517 EPIISL418518 EPIISL418519 EPIISL418520 EPIISL418574 EPIISL418576 EPIISL418579 EPIISL421096 EPIISL421101 EPIISL421102 EPIISL421105 EPIISL421109 EPIISL421111 EPIISL424746 EPIISL428211 EPIISL430757 EPIISL435318 EPIISL435323 EPIISL435324 EPIISL443120 EPIISL443121 EPIISL443123 EPIISL443124 EPIISL443125 EPIISL443126 EPIISL443129 EPIISL443130 EPIISL443131 EPIISL443132 EPIISL443133 EPIISL443134 EPIISL443136 EPIISL443137 EPIISL443138 EPIISL443139 EPIISL491489 EPIISL491761 EPIISL491763 EPIISL491764 EPIISL491767 EPIISL491768 EPIISL491769 EPIISL491771 EPIISL491772 EPIISL491773 EPIISL491775 EPIISL491778 EPIISL491779 EPIISL491783 EPIISL491784 EPIISL491785 EPIISL491787 EPIISL491788 EPIISL491794 EPIISL491795 EPIISL491796 EPIISL491798 EPIISL491802 EPIISL491804 EPIISL491805 EPIISL491806 EPIISL491807 EPIISL491808 EPIISL491809 EPIISL491810 EPIISL491811 EPIISL491814 EPIISL491815 EPIISL491818 EPIISL491820 EPIISL491821 EPIISL491822 EPIISL491824 EPIISL491826 EPIISL491827 EPIISL491830 EPIISL491832 EPIISL491833	168
141.	**5562**	PB1, PA	1		
142.	**3519**	PA, NP	0.99		
143.	**4058**	PA, N2	0.99		
144.	**8676**	PB1, NS	0.99	EPIISL363896 EPIISL363898	2
145.	**5409**	PB1, MP	0.99		
146.	**4050**	N2, MP	1		
147.	**9136**	H3, PB2	0.99		
148.	**8223**	H3, PB1	1		
149.	**7172**	H3, PA	0.99		
150.	**9829**	H3, N2	1		
151.	**4907**	PB2, PA	1	EPIISL363957 EPIISL363958 EPIISL363960 EPIISL363963 EPIISL363964 EPIISL363988 EPIISL366007	7
152.	**4719**	PB2, N2	1		
153.	**4813**	PB1, PA	1		
154.	**4618**	PB1, N2	1		
155.	**4969**	PA, NP	0.99		
156.	**3489**	PA, MP	0.99		
157.	**2963**	N2, MP	1		
158.	**3350**	H3, PA	1		
159.	**3095**	H3, NS	0.99		
160.	**4734**	H3, N2	1		
161.	**2369**	PB2, NS	0.99	EPIISL365773	1
162.	**2754**	PB1, NS	1		
163.	**2280**	PA, NS	0.99		
164.	**2948**	NP, NS	0.99		
165.	**3654**	N2, NS	0.99		
166.	**1912**	MP, NS	1		
167.	**2399**	H3, NS	0.99		
168.	**6217**	PB2, PB1	1	EPIISL365784 EPIISL365785 EPIISL390030 EPIISL393570	4
169.	**4700**	PB2, PA	1		
170.	**5232**	PB1, NP	1		
171.	**823**	PB1, MP	1		

172.	**2973**	PA, NP	1		
173.	**4547**	NP, N2	1		
174.	**5599**	H3, PB2	1		
175.	**3321**	H3, NP	1		
176.	**775**	H3, MP	1		
177.	**5717**	PB2, N2	1	EPIISL365784 EPIISL365785 EPIISL390030 EPIISL393570 EPIISL400779	5
178.	**5001**	NP, N2	0.99		
179.	**3156**	N2, MP	0.99		
180.	**2032**	PB1, MP	1	EPIISL393689	1
181.	**1826**	PA, MP	0.99		
182.	**1767**	NP, MP	0.99		
183.	**1639**	N2, MP	1		
184.	**1650**	MP, NS	0.99		
185.	**1730**	H3, MP	1		
186.	**12740**	PB2, MP	0.99	EPIISL393689 EPIISL394106 EPIISL394110	3
187.	**13094**	PA, MP	1		
188.	**10393**	NP, MP	0.99		
189.	**1058**	PB2, MP	1	EPIISL393689 EPIISL394110	2
190.	**1034**	PA, MP	1		
191.	**1035**	NP, MP	1		
192.	**1277**	PB2, PA	0.99	EPIISL393752	1
193.	**1417**	PB1, PA	1		
194.	**1024**	PA, NP	0.99		
195.	**2170**	H3, PB2	1		
196.	**1778**	H3, PB1	1		
197.	**1629**	H3, NS	0.99		
198.	**1009**	H3, NP	1		
199.	**2361**	H3, N2	1		
200.	**1273**	H3, MP	0.99		
201.	**2070**	PB2, PA	1	EPIISL393752 EPIISL393968	2
202.	**2556**	PB1, PA	1		
203.	**11944**	PA, NP	0.99		
204.	**15367**	PA, N2	1		
205.	**930**	PB2, NS	1	EPIISL393963	1
206.	**13894**	PB1, NS	1		
207.	**1024**	PA, NS	1		
208.	**844**	NP, NS	1		
209.	**13619**	N2, NS	1		
210.	**1413**	H3, NS	1		
211.	**1011**	PB2, NS	0.99	EPIISL393965	1
212.	**843**	PB1, NS	1		
213.	**539**	NP, NS	1		
214.	**1114**	N2, NS	1		
215.	**1326**	H3, NS	1		
216.	**12920**	PB2, NS	0.99	EPIISL393965 EPIISL394110	2
217.	**16058**	PA, NS	0.99		
218.	**16008**	H3, NS	0.99		
219.	**1643**	PB2, PB1	1	EPIISL393966	1
220.	**945**	PB2, PA	1		
221.	**819**	PB2, NS	1		
222.	**820**	PB2, NP	1		
223.	**1615**	PB2, N2	1		
224.	**979**	PB2, MP	0.99		
225.	**1951**	H3, PB2	1		
226.	**944**	PB2, PA	1	EPIISL393968	1
227.	**1167**	PB1, PA	1		
228.	**914**	PA, NS	1		
229.	**597**	PA, NP	1		
230.	**820**	PA, N2	1		
231.	**1207**	H3, PA	1		
232.	**1546**	PB2, PB1	0.99	EPIISL394106	1
233.	**882**	PB2, PA	1		
234.	**788**	PB2, NS	1		
235.	**1559**	PB2, N2	1		
236.	**911**	PB2, MP	1		
237.	**1101**	PB1, PA	0.99		
238.	**1791**	H3, PB2	1		
239.	**779**	PB1, NS	0.99	EPIISL400779	1
240.	**1232**	PB1, N2	1		
241.	**1192**	PA, NS	0.99		
242.	**1159**	PA, N2	1		
243.	**2959**	NP, NS	0.99		
244.	**3180**	NP, N2	1		
245.	**953**	H3, NS	1		
246.	**1618**	H3, N2	1		
247.	**295**	PB2, PB1	1	FTL1393	1
248.	**274**	PB1, PA	1		
249.	**168**	PB1, NS	1		
250.	**164**	PB1, NP	1		
251.	**139**	PB1, N2	1		
252.	**411**	H3, PB2	1		
253.	**299**	H3, PA	1		
254.	**349**	H3, NS	1		
255.	**212**	H3, NP	1		
256.	**319**	H3, N2	1		

Supplementary Table S6: Intra-subtype reassortment events and reassortants identified by GiRaF among the 1224 H3N2 virus whole genomes sampled in 2009-2020 in Africa.

Supplementary Fig. S7: Variation in the number of H1N1pdm09 reassortants and whole genomes sampled

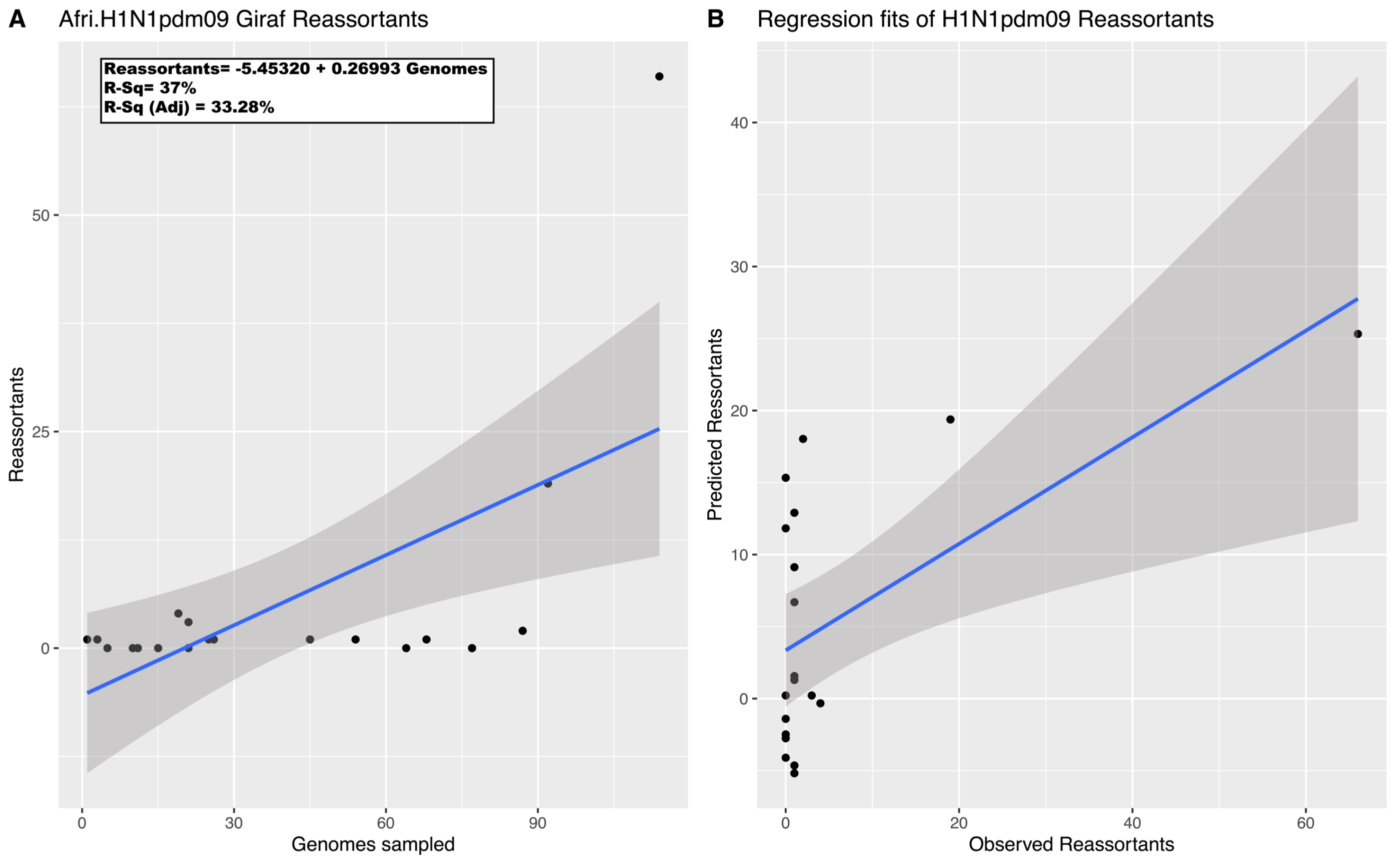

Supplementary Fig. S7: Variation in the number of reassortants observed with number of whole genomes sampled among Africa H1N1pdm09 viruses. Panel A shows the variation of number of reassortants observed with number of whole genomes sampled or analysed in 19 African countries. **Panel B** shows the number of reassortants predicted by the linear regression model against the number of reassortants observed.

Supplementary Fig. S8: Variation in the number of H3N2 reassortants and whole genomes sampled

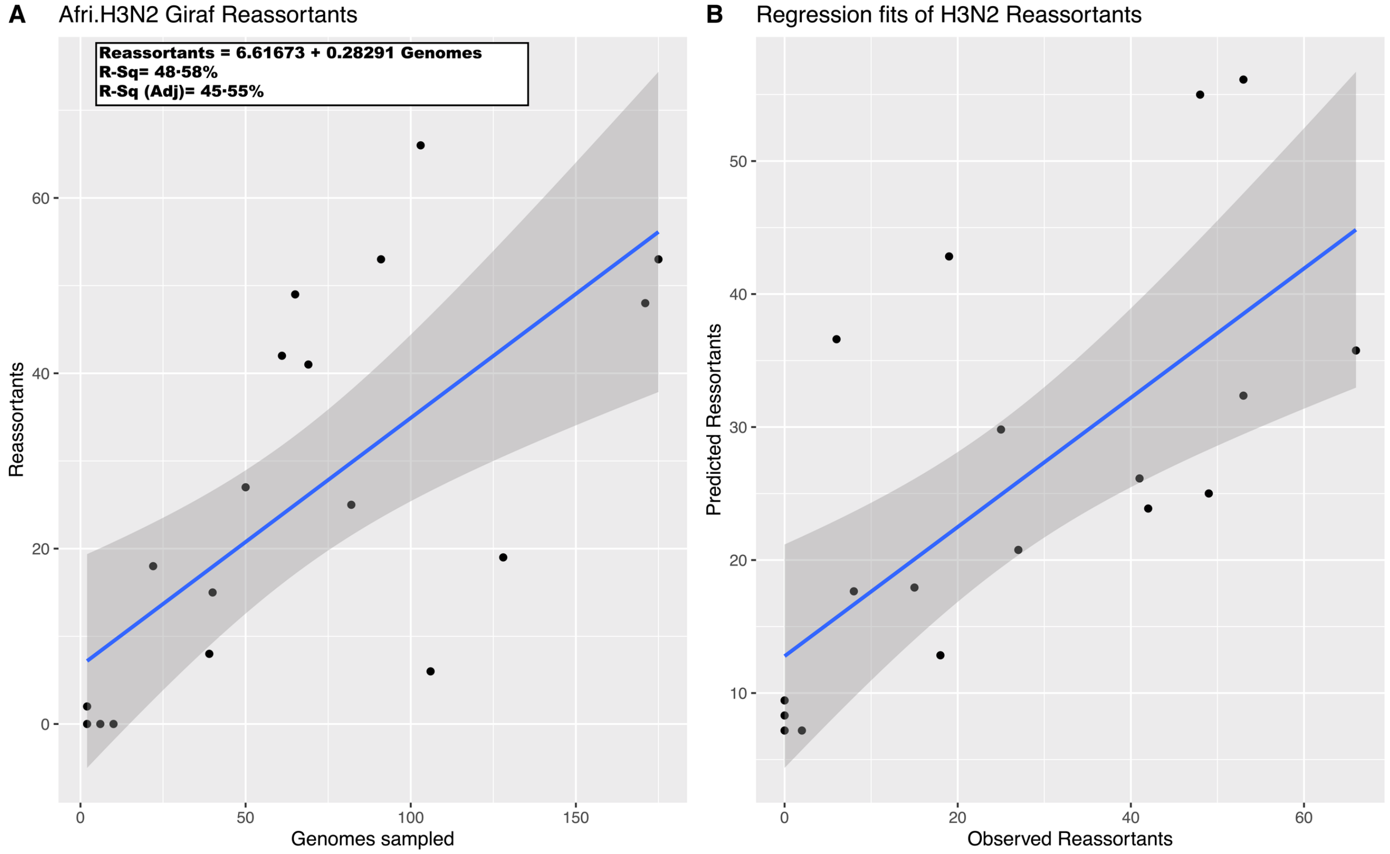

Supplementary Fig. S8: Variation in the number of reassortants observed with number of whole genomes sampled among Africa H3N2 viruses. Panel A shows the variation of number of reassortants observed with number of whole genomes sampled or analysed in 19 African countries. **Panel B** shows the number of reassortants predicted by the linear regression model against the number of reassortants observed.
